# Advancements in Carbazole-Based Sensitizers and Hole-Transport Materials for Enhanced Photovoltaic Performance

**DOI:** 10.3390/molecules29215035

**Published:** 2024-10-25

**Authors:** Ayagoz Ibrayeva, Urker Abibulla, Zulfiya Imanbekova, Bakhytzhan Baptayev, Robert J. O’Reilly, Mannix P. Balanay

**Affiliations:** 1 National Laboratory Astana, Nazarbayev University, 53 Kabanbay Batyr Ave., Astana 010000, Kazakhstanbbaptayev@nu.edu.kz (B.B.); 2 Department of Chemistry, L.N. Gumilyov Eurasian National University, 2 Satpayev St., Astana 010008, Kazakhstan; 3 Chemistry Department, Nazarbayev University, 53 Kabanbay Batyr Ave., Astana 010000, Kazakhstan; 4 School of Science and Technology, University of New England, Armidale, NSW 2351, Australia

**Keywords:** carbazole-based dyes, donor-pi-acceptor, metal-free organic sensitizer, hole-transport materials

## Abstract

Carbazole-based molecules play a significant role in dye-sensitized solar cells (DSSCs) due to their advantageous properties. Carbazole derivatives are known for their thermal stability, high hole-transport capability, electron-rich (p-type) characteristics, elevated photoconductivity, excellent chemical stability, and commercial availability. This review focuses on DSSCs, including their structures, working principles, device characterization, and the photovoltaic performance of carbazole-based derivatives. Specifically, it covers compounds such as 2,7-carbazole and indolo[3,2-b]carbazole, which are combined with various acceptors like benzothiadiazole, thiazolothiazole, diketopyrrolopyrrole, and quinoxaline, as reported over the past decade. The review will also outline the relationship between molecular structure and power-conversion efficiencies. Its goal is to summarize recent research and advancements in carbazole-based dyes featuring a D-π-A architecture for DSSCs. Additionally, this review addresses the evolution of carbazole-based hole-transport materials (HTMs), which present a promising alternative to the costly spiro-OMeTAD. We explore the development of novel HTMs that leverage the unique properties of carbazole derivatives to enhance charge transport, stability, and overall device performance. By examining recent innovations and emerging trends in carbazole-based HTMs, we provide insights into their potential to reduce costs and improve the efficiency of DSSCs.

## 1. Introduction

The global demand for a sustainable energy supply has never been more pressing. As the need for alternative energy sources grows, advancements in solar cell technology have emerged. Although commercially available single- and multi-crystalline silicon and inorganic solar cells are highly efficient at converting sunlight into electricity, they pose environmental challenges due to the use of toxic chemicals like trichlorosilane and silicon tetrachloride, as well as high production costs. Consequently, a new generation of solar cells, such as organic photovoltaics (OPVs), perovskite solar cells (PSCs), and dye-sensitized solar cells (DSSCs), is being explored. These alternatives offer lower manufacturing costs, greater mechanical flexibility, and improved color selectivity. Among these, DSSCs have garnered significant attention for their potential to power both outdoor and indoor applications, thus offering a versatile solution for various electrical devices [1,2,3].

Proper design and synthesis play a crucial role in advancing innovative organic functional materials for optoelectronic applications, including organic light-emitting diodes (OLEDs), photovoltaics, non-linear optics (NLOs), field-effect transistors (OFETs), and sensors [4]. One such material is a carbazole-based self-assembled monolayer (SAM) containing phosphonic acid known as 2PACz, which has proven effective as hole-transport material (HTM) (HTLs) in perovskite light-emitting diodes (PeLEDs), organic solar cells, and perovskite solar cells, demonstrating exceptional performance [5]. Electrochemical polymerization is widely employed for creating thin films of carbazole-based polymers [6]. Understanding the electrochemical behavior of carbazole and its derivatives is crucial for developing novel materials and devices with desirable properties, such as stability [7]. Carbazole derivatives with diallyl substitutions are particularly effective as host materials for high-efficiency phosphorescent OLEDs in the blue, green, and red color spectrum [8]. Recent reviews highlight the role of carbazole derivatives as donors in combination with various acceptor groups in bulk heterojunction OSCs, DSSCs, and PSCs [9]. Advances in molecular design, hole-transporting properties, power-conversion efficiency (PCE), and thermal stability of organic hole-transporting materials based on different carbazole derivatives have been extensively investigated for PSC applications [10]. Significant research efforts have also been dedicated to the utilization of carbazole derivatives as HTMs in both PSCs [11] and OLEDs [5,12].

In comparison to other photovoltaic technologies like PSCs, which have a higher power-conversion efficiency (PCE) of 26.1% for single-junction devices [13,14,15], DSSCs stand out due to their lower production costs, better low-light performance, shorter energy-payback times, enhanced environmental sensitivity, and relative ease of fabrication. Initially developed in 1991 by O’Regan and Grätzel using a Ru(II) pyridyl complex dye and an I^−^/I_3_^−^ electrolyte, DSSCs achieved a PCE of 7.9% [16]. Recent advancements have increased the highest reported efficiency for DSSCs to 13.6% with a single dye [17]. However, the use of a co-sensitization approach, in which multiple dyes are employed, has allowed for the rise of DSSCs with PCEs of up to 15.2% [18]. In low-light conditions, DSSCs have a significant advantage over other third-generation photovoltaic technologies due to their simpler chemical composition. Utilizing a co-sensitization process with a copper (II/I) electrolyte, DSSCs have achieved an efficiency of 34% at 1000 lux [19]. Since the theoretical efficiency limits are 33% for AM 1.5 G illumination and 57% for various indoor lighting conditions [20], there is significant potential to enhance the efficiency and versatility of DSSCs. Improvements in module design, substrate integration, architecture, reliability, and adaptable manufacturing methods could greatly advance DSSCs. This potential has sparked considerable research interest and opened promising avenues for commercialization.

Over the last decade, there has been a growing emphasis on hole-transport materials, yet the synthesis, classification, and application of organic HTMs using carbazole in dye-sensitized solar cells have received comparatively little attention. Effective hole transport facilitated by HTMs ensures that generated holes can swiftly reach the anode without significant loss through electron recombination. Proper alignment of energy levels between the HTM, dye, and anode is crucial for efficient charge transfer. A well-chosen HTM contributes to the long-term stability of DSSCs by averting performance degradation. Carbazole derivatives are particularly suitable for HTMs due to their high hole mobility and adaptable electrical properties.

Organic photovoltaic cells face a significant drawback due to their lower charge-carrier mobilities compared to traditional inorganic semiconductors. This limitation is underscored by the dispersive nature of charge-carrier mobility in organic semiconductors, which directly impacts critical processes such as charge-transfer state dissociation, free-charge extraction, and recombination—key factors influencing the efficiency of organic photovoltaics. Recent advancements delved into the influence of charge-carrier mobility on these efficiency-defining mechanisms [21]. In contrast, DSSCs exhibit a distinct advantage with their separation of light absorption and charge-carrier transport activities, enabling more effective light harvesting [22]. Understanding and optimizing charge-carrier mobility is crucial as it influences processes like exciton migration, dissociation, and charge collection at electrodes, pivotal for enhancing solar cell performance [23]. The diffusion model is increasingly favored for simulating charge-carrier densities in DSSCs due to its ability to capture complex transport phenomena [24]. Recent developments in DSSC efficiency focus on improved electrodes, enhanced carrier-transport materials, and molecular engineering [25], aiming to address challenges in scaling up from laboratory to industrial applications to improve efficiency and stability. Additionally, incorporating hexyloxyphenyl substituents has shown promise in enhancing electron lifetime and open-circuit voltage by blocking I_3_^−^ ions in the electrolyte [26]. The molecular design also plays a crucial role in optimizing carbazole-based sensitizers for improved electron transfer in DSSCs, with properties like polarizability significantly influencing charge-transfer processes and overall efficiency [27]. These insights underscore the importance of advancing both material science and molecular engineering to propel the efficiency and viability of organic and dye-sensitized solar cells.

This review aims to fill a gap in the literature by exploring carbazole-based hole-transport materials (HTMs) and the application of carbazole-based dyes as sensitizers. It will examine the synthesis and fabrication of these materials, along with their electrochemical and photovoltaic characterization, as well as density functional theory (DFT) studies.

## 2. Working Principle of DSSCs

**Figure 1:**
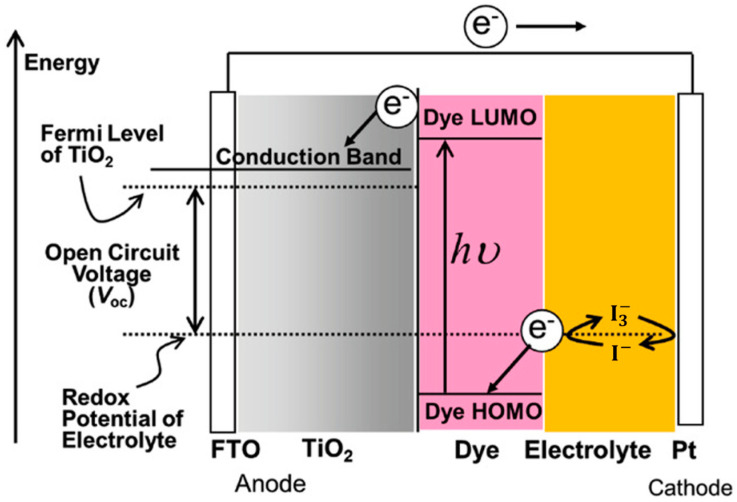
Dye-sensitized solar cell structure and energy diagram. Adapted with permission from reference [3]. Copyright (2018), John Wiley and Sons.

Figure 1 illustrates the general structure of DSSCs, which consist of a dye-sensitized mesoporous photoanode (a film of dye-anchored nanocrystaline titanium dioxide, TiO_2_), an electrolyte (generally a I^−^/I_3_^−^ redox mediator, though Co^2^⁺/Co^3^⁺ or Cu^+^/Cu^2+^ are also used), and a counter electrode (transparent conductive oxide glass treated with platinum). Sunlight is absorbed by a monolayer of sensitizer attached to the surface of the TiO_2_ semiconductor, exciting electrons from the highest occupied molecular orbital (HOMO) to the lowest unoccupied molecular orbital (LUMO). These excited electrons then diffuse from the conductive band of TiO_2_ to the platinized counter electrode via an external electrical circuit. The photoelectric cycle is completed when the oxidized dye is regenerated by the I⁻ species, forming I_3_^−^, which then diffuses to the counter electrode and is reduced [1,2,3].

For efficient solar cell operation, each component must perform optimally. The counter electrode needs to have high catalytic activity and stability. While alternative materials like transition metal compounds, polymers, and carbon-based materials show potential, they are not yet thoroughly characterized [28]. Consequently, platinum remains the most efficient, though its degradation upon contact with iodine redox electrolytes, high cost, and scarcity limit its commercial viability.

The electrolyte, which regenerates the oxidized sensitizer and facilitates charge transfer between the counter electrode and the photoanode, must also offer long-term stability. Traditional liquid iodine redox electrolytes and other ionic conductivity-based alternatives face issues such as solvent evaporation, degradation of the dye and counter electrode, and difficulties with cell sealing. Polymer electrolytes address these issues, enhancing cell longevity.

Finally, sensitizers, which are crucial to DSSCs, must meet several criteria: They should absorb a wide spectrum from visible to near-infrared light efficiently, possess stable HOMO and LUMO energy levels to facilitate charge transfer to TiO_2_, resist degradation, retain light-harvesting capability for at least 20 years, and attach effectively to the semiconductor oxide film with an anchoring group while being regenerated by the electrolyte [1,29].

## 3. Carbazole-Based Dyes for DSSCs

**Figure 2:**
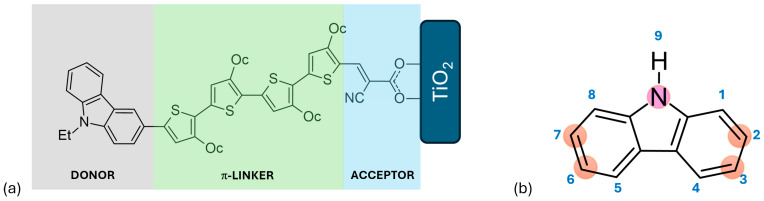
(**a**) Schematic representation of a D-π-A structure featuring a carbazole-based dye, where Et denotes ethyl and Oc denotes octyl groups. (**b**) Structural depiction of carbazole, highlighting atom numbering and the positions most commonly substituted.

Dyes can have a range of structural architectures, including D-A, D-π-A, D-D-π-A, D-A-π-A, and D-π-D-π-A, A-π-D-π-A, A-π-D-π-A-π-A, D-π-D-A, D-(π-D-A)_2_, D-(π-D-A)_3_, among others. In these structures, “D” represents the donor moiety, “π” signifies the π-linker or π-bridge, and “A” denotes the acceptor or anchoring group, which is typically a carboxylic acid or cyanoacrylic acid. The most conventional and studied structure is the D-π-A configuration, depicted in Figure 2a. This straightforward structure can be modified in numerous ways to enhance its effectiveness, making it particularly suitable for research [30]. Notably, the D-π-A design is highly effective for metal-free organic sensitizers because it offers a broad and strong optical absorption band in the visible spectrum thanks to the efficient intramolecular charge transfer (ICT) from the donor to the acceptor. Upon light irradiation, electrons are excited from the donor’s highest-occupied molecular orbital to the acceptor’s lowest unoccupied molecular orbital and then transferred to the acceptor, which is attached to the TiO_2_ substrate via a strong ionic bond. This arrangement promotes efficient electron injection into the conductive band of the TiO_2_ semiconductor oxide film [1,31].

Numerous compounds with a D-π-A structure have been utilized as sensitizers, including coumarin, triarylamine, carbazole, phenothiazine, diketopyrrolopyrrole, and triphenylamine, among others [32,33,34,35]. Carbazole, illustrated in Figure 2, is particularly noteworthy due to its electron-rich properties, excellent hole-transport capabilities, commercial availability, and remarkable chemical and thermal stability. Its electron-rich nature makes it suitable for use as an auxiliary donor, donor, or π-bridge. Furthermore, carbazole can be easily functionalized at the 2, 7, 3, 6, and 9 positions, as depicted in Figure 2b [1,3]. Prior to 2011, the primary applications of carbazole-based functional materials were as difunctionalized derivatives capable of transporting or emitting holes and serving as donors in donor–acceptor molecular topologies. Recently, the intriguing structure–function correlations resulting from the polyfunctionalization of carbazole at various locations have garnered significant attention [4].

This literature review provides an overview of recent research on carbazole-based dyes with a D-π-A architecture, as well as a compilation of various architectural structures of these dyes. The focus is specifically on their applications in dye-sensitized solar cells (DSSCs), as detailed in Table 1, Table 2, Table 3, Table 4 and Table 5. The review investigates how the structural design of carbazole-based sensitizers influences the performance of these devices. These dyes have demonstrated efficiencies comparable to the widely used commercial N719 dye [36], suggesting their potential for commercial use, contingent upon enhancements in physical, chemical, and stability properties. The review underscores the promise of carbazole-based dyes, highlighting their photovoltaic characteristics to stimulate further research interest among scholars and scientists. In DSSCs, the cell’s performance is evaluated using key parameters such as short-circuit current density (J_sc_) and open-circuit voltage (V_oc_). The formulas for these parameters are as follows: The short-circuit current density measured in units of mA/cm^2^ or A/m^2^ is determined using the formula:

(1) $$J_{SC}=\frac{I_{SC}}{A}$$

Here, I_SC_ represents the short-circuit current, which is the total current when the solar cell is shorted, provided in mA or A, and A denotes the area of the solar cell in cm^2^ or m^2^. Meanwhile, the open-circuit voltage, measured in volts (V), can be calculated using the formula:

(2) $$V_{OC}=\frac{kT}{q}\ln\frac{J_{SC}}{J_{o}}+1$$

In this equation, k is Boltzmann’s constant (1.38 × 10^−23^ J/K), T is the absolute temperature in Kelvin, q is the charge of an electron (1.6 × 10^−19^ C), and J_o_ is the saturation-current density, which is the current density at zero voltage, measured in A/m^2^.

These equations elucidate the critical performance metrics of solar cells, crucial for optimizing the efficiency and viability of carbazole-based dye applications in photovoltaic technologies.

### 3.1. Recent Developments on Carbazole-Based Dyes

#### 3.1.1. D-π-A Architecture

**Figure 3:**
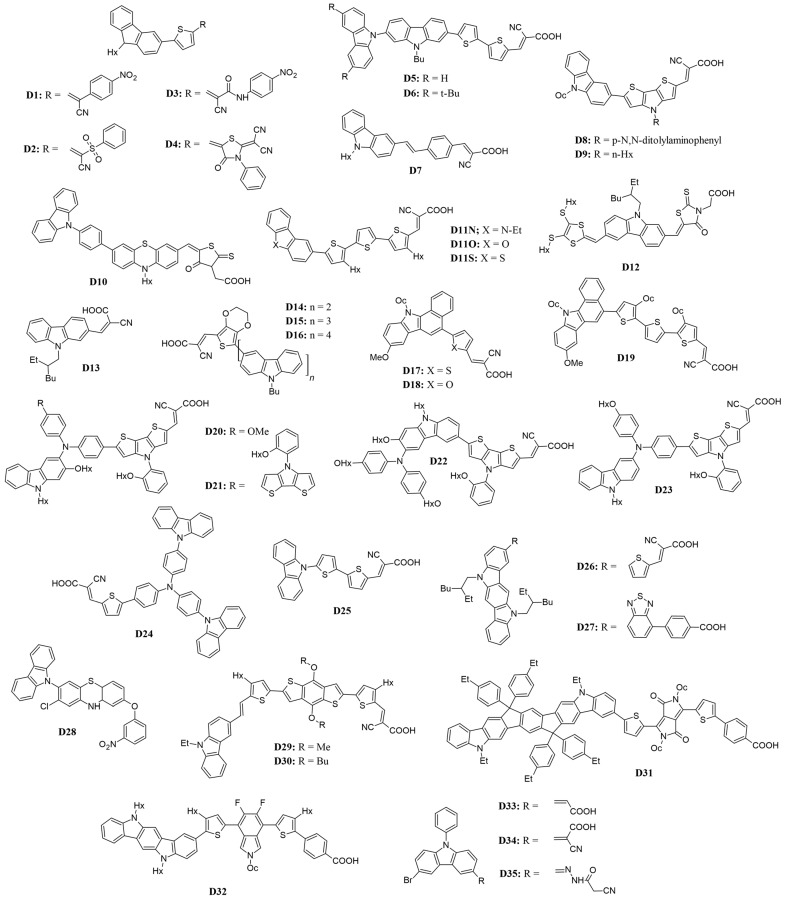
Structure of the carbazole-based molecules with D-π-A architecture (**D1** to **D35**) as sensitizers for DSSCs.

To broaden the absorption spectra of the D-π-A system through molecular engineering, two primary strategies are employed: increasing the effective conjugation length between the donor and acceptor and enhancing the electron-donating or electron-withdrawing capabilities, as presented in Table 1 and Figure 3. Introducing alkyl chains is a common approach to modifying molecular properties. Alkyl chains can enhance the electron-donating ability of carbazole, influencing its HOMO levels and overall charge-transport characteristics. Additionally, longer or branched alkyl chains can improve solubility in organic solvents, facilitating device fabrication, while they also help prevent π-aggregation, which enhances performance metrics such as charge injection and collection efficiency [37,38].

**Table 1:** Photovoltaic parameters of the DSSCs based on carbazole-based sensitizers having D-π-A architecture.

Dye No.	Electrolyte	λ_max_ nm, (ε × 10^4^ M^−1^ cm^−1^)	PCE (%)	FF	V_OC_ (V)	J_SC_ (mA cm^−2^)	Refs.
**D1**	I^−^/I_3_^−^	495 (5.13) ^a^	6.06	0.75	0.56	14.43	[39]
**D2**	I^−^/I_3_^−^	491 (5.39) ^a^	7.39	0.66	0.70	16.00	[39]
**D3**	I^−^/I_3_^−^	522 (4.27) ^a^	8.38	0.69	0.72	16.88	[39]
**D4**	I^−^/I_3_^−^	499 (3.15) ^a^	9.55	0.70	0.78	17.50	[39]
**D5**	I^−^/I_3_^−^	476 (4.51) ^b^	6.04	0.64	0.601	15.78	[40]
**D6**	I^−^/I_3_^−^	478 (4.47) ^b^	5.48	0.64	0.612	14.00	[40]
**D7**	I^−^/I_3_^−^ Co^3+^/Co^2+^	435 (3.42) ^c^	7.10 9.00	0.65 0.67	0.645 0.725	17.20 18.30	[41]
**D8**	I^−^/I_3_^−^	492 (5.64) ^d^	6.63	0.64	0.71	14.55	[42]
**D9**	I^−^/I_3_^−^	496 (6.25) ^d^	6.50	0.63	0.70	14.71	[42]
**D10**	I^−^/I_3_^−^	493 (1.89) ^b^	5.57	0.52	0.71	15.12	[43]
**D11N**	I^−^/I_3_^−^	492 (2.43) ^e^	7.44	0.67	0.701	15.73	[44]
**D11O**	I^−^/I_3_^−^	476 (2.69) ^e^	7.68	0.69	0.714	15.61	[44]
**D11S**	I^−^/I_3_^−^	477 (2.78) ^e^	7.18	0.67	0.691	15.43	[44]
**D12**	I^−^/I_3_^−^	551 (1.15) ^a^	7.38	0.67	0.78	14.12	[45]
**D13**	I^−^/I_3_^−^	552 (1.01) ^a^	6.90	0.69	0.76	13.19	[45]
**D14**	I^−^/I_3_^−^	433 (1.46) ^e^	6.16	0.694	0.706	12.51	[46]
**D15**	I^−^/I_3_^−^	449 (1.07) ^e^	7.21	0.672	0.723	14.84	[46]
**D16**	I^−^/I_3_^−^	447 (1.17) ^e^	6.97	0.683	0.707	14.44	[46]
**D17**	I^−^/I_3_^−^	458 (2.02) ^b^	6.01	0.660	0.729	12.40	[47]
**D18**	I^−^/I_3_^−^	462 (2.15) ^b^	6.93	0.660	0.757	13.80	[47]
**D19**	I^−^/I_3_^−^	477 (3.24) ^b^	7.54	0.680	0.744	14.80	[47]
**D20**	I^−^/I_3_^−^ Co^3+^/Co^2+^	535 (4.71) ^b^	7.80 8.67	0.690 0.680	0.754 0.880	15.00 14.50	[48]
**D21**	I^−^/I_3_^−^ Co^3+^/Co^2+^	518 (3.81) ^b^	5.43 4.65	0.680 0.690	0.650 0.763	12.30 8.80	[48]
**D22**	I^−^/I_3_^−^ Co^3+^/Co^2+^	517 (5.39) ^b^	6.73 7.06	0.690 0.680	0.723 0.845	13.50 12.30	[48]
**D23**	I^−^/I_3_^−^ Co^3+^/Co^2+^	511 (4.04) ^b^	6.27 6.77	0.670 0.680	0.709 0.830	13.20 12.00	[48]
**D24**	I^−^/I_3_^−^	442 (2.95) ^f^	5.92	0.589	0.740	13.6	[49]
**D25**	I^−^/I_3_^−^	435 (1.65) ^g^	5.10	0.730	0.720	9.89	[50]
**D26**	I^−^/I_3_^−^	500 (5.48) ^b^	5.41	0.590	0.745	12.55	[51]
**D27**	I^−^/I_3_^−^	463 (1.58) ^b^	5.01	0.660	0.744	10.16	[51]
**D28**	I^−^/I_3_^−^	442 ^a^	5.68	0.761	0.726	11.69	[52]
**D29**	Co^3+^/Co^2+^	492 (3.39) ^h^	5.27	0.620	0.775	11.05	[53]
**D30**	Co^3+^/Co^2+^	492 (3.35) ^h^	5.60	0.600	0.783	11.93	[53]
**D31**	Cu^2+^/Cu^+^	625 (4.32) ^b^	6.00	0.754	0.95	8.30	[54]
**D32**	Cu^2+^/Cu^+^	422 (5.08) ^b^	5.10	0.647	1.100	7.10	[54]
**D33**	I^−^/I_3_^−^	478 ^i^	6.72	0.690	0.780	11.18	[55]
**D34**	I^−^/I_3_^−^	466 ^i^	7.46	0.710	0.810	13.57	[55]
**D35**	I^−^/I_3_^−^	481 ^i^	8.12	0.680	0.850	14.72	[55]

Note: A Pt electrode was utilized as a counter electrode. Absorption maxima in ^a^ THF, ^b^ DCM, ^c^ EtOH, ^d^ CH_2_Cl_2_/THF, ^e^ CHCl_3_, ^f^ MeCN, ^g^ CH_2_Cl_2_, ^h^ 1:1 toluene:MeCN, and ^i^ DMSO solutions.

NMR spectroscopy is essential for characterizing carbazole derivatives and confirming the successful introduction of alkyl chains. The protons on the alkyl chain typically resonate between 0.9 ppm (for methyl groups) and 1.5 ppm (for methylene groups), while the protons on the carbazole ring appear around 7–8 ppm due to their aromatic nature. Moreover, IR spectroscopy offers valuable insights into the functional groups present in carbazole derivatives. For instance, C–H stretching vibrations from alkyl chains are found in the range of 2800–3000 cm^−1^, while aromatic C=C stretching vibrations are typically observed around 1500–1600 cm^−1^. The absence of strong peaks near 1700 cm^−1^ suggests a lack of carbonyl functionalities, indicating that only alkyl groups are present.

**Figure 4:**
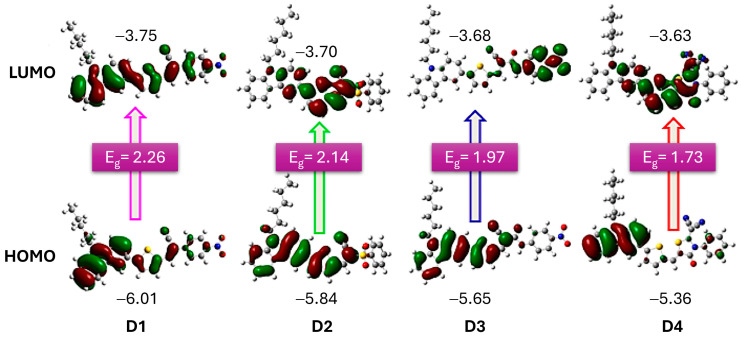
Optimized structures of dyes D1 to D4, illustrating their HOMO and LUMO frontier molecular orbitals. The accompanying values indicate the HOMO and LUMO energy levels, along with the calculated band gap (E_g_). Adapted with permission from reference [39]. Copyright (2023), Elsevier.

In a study by Elmorsy et al. [39], four carbazole-based organic sensitizers (**D1**–**D4**) were synthesized, demonstrating efficiencies ranging from 6.06% to 9.55% and a broad light-harvesting range from 400 to 800 nm. Notably, the power-conversion efficiency of the **D4** dye reached 9.55%, representing a 32% increase compared to the N719 sensitizer, attributed to the incorporation of a thiazolidine-4-one ring that enhances electron injection and reduces recombination. The energy bandgap (E_g_) of these organic sensitizers was calculated using the difference in energy levels (E_HOMO_–E_LUMO_), revealing a decrease in E_g_ values from **D1** to **D4**. Specifically, **D4**, with its elongated π-conjugated linker due to the new thiazolidine-4-one acceptor, exhibits the smallest bandgap, allowing it to absorb lower-energy photons with longer wavelengths. This characteristic facilitates the excitation of the sensitizer’s electrons into unoccupied π orbitals, shown in Figure 4, thereby enhancing the stability, short-circuit current density, and overall PCE of solar cells.

Venkateswararao et al. [40] introduced a series of new organic dyes (**D5**–**D6**) featuring a carbazole/3,6-di(*tert*-butyl) carbazole donor, a 2,7-carbazole spacer, and cyanoacrylic acid as both the acceptor and anchoring group. Compared to similar dyes with 3,6-carbazole or phenyl linkers, these dyes demonstrated enhanced light-harvesting capabilities. Specifically, the open-circuit voltage (V_OC_) values for thiophene derivatives (0.635 V and 0.666 V) were higher than those for their bithiophene counterparts **D5** and **D6** (0.601 V and 0.612 V) due to a more positive LUMO level. Additionally, dyes with *tert*-butyl substituents (**D6**) exhibited higher V_OC_ values than their unsubstituted counterparts (**D5**), owing to the improved light-harvesting properties and a hydrophobic environment that reduces electrolyte contact with the photoanode. The PCE of the bithiophene-based dyes (6.04% and 5.48%) was greater than that of the thiophene-based dyes (4.22% and 4.95%), attributed to superior absorption properties and electron-collection efficiency.

To evaluate the impact of the anchoring group, Soni et al. [41] synthesized two additional carbazole-based dyes. These dyes used carbazole as the donor and cyanoacrylic acid (**D7**) or rhodamine-3-acetic acid as the acceptor, connected by a vinylene-phenylene π bridge. The dyes achieved efficiencies of 2.0% for rhodamine-3-acetic acid–acceptor and 7.1% for **D7** with an iodine redox electrolyte, as well as 2.4% and 9.0% with a cobalt (II/III) redox electrolyte. The dye with the cyanoacrylic acid acceptor group outperformed the one with the rhodamine-3-acetic acid group, owing to more effective electron injection into the conduction band of TiO_2_. This is due to better overlapping with the surface and reduced charge recombination between the injected electron and the oxidized electrolyte.

Zhang and colleagues [42] introduced two innovative dyes (**D8**–**D9**) featuring an alkylated carbazole donor, dithienopyrrole (DTP) as a π-bridge, and cyanoacetic acid as the acceptor. The dye with a benzene ring attached to the DTP showed a higher PCE of 6.63% compared to the alkylated DTP dye, which had a PCE of 6.50%. The former also had a higher V_OC_ of 0.71 V versus 0.70 V for the latter. This improvement is attributed to reduced π-π aggregation of the dyes on TiO_2_ films and minimized charge recombination.

In another research, the group of Jiao et al. [43] evaluated the impact of the *meta*- and *para*-positions of donors in the dye structure (**D10**). In their carbazole, it served as the donor, connected to a phenothiazine spacer through a benzene ring, with rhodamine acetic acid acting as the acceptor and anchoring group. The dye with carbazole connected at the *meta* position exhibited a lower PCE of 4.56% compared to the *para*-connected dye (**D10**), which had a PCE of 5.57%. The *para*-connected carbazole showed a superior short-circuit current density of 15.12 mA cm^−2^ due to higher dye loading, more effective ICT, and a broader absorption spectrum. Additionally, the para-connected carbazole’s lower charge-recombination resistance contributed to a longer electron lifetime and lower transport-charge resistance, enhancing dye stability.

A series of dyes (**D11N**, **D11O**, and **D11S**) incorporating heteroatom donors and cyanoacetic acid acceptors linked by alkyl-substituted thiophene units have also been studied [44]. Based on the results of this study, it was shown that the *O*-substituted dibenzo[b,d]furan-2-yl motif (**D11O**) achieved the highest conversion efficiency of 7.68%. In addition, this study demonstrated that the *N*-substituted carbazole motif (**D11N**) exhibited a better power-conversion efficiency (7.44%) when compared to the *S*-substituted dye (**D11S**, which is based on a dibenzo[b,d]thiophen-2-yl motif), which had a PCE of 7.18%. This performance difference is attributed to the greater electron-donating ability of carbazole compared to dibenzothiophene. Furthermore, introducing alkyl chains to the spacer reduced dye aggregation on the semiconductor oxide film, allowing planar dyes to achieve high V_OC_ values (0.701 V, 0.714 V, and 0.691 V for **D11N**, **D11O**, and **D11S**, respectively).

Duvva and Giribabu [45] introduced new dyes featuring a carbazole π-bridge that links a donor dithiafulvalene unit with acceptors cyanoacrylic acid (**D12**) or rhodanine-3-acetic acid (**D13**). Devices incorporating cyanoacrylic acid-based dyes achieved the highest efficiency of 7.38%, compared to 6.90% for those using rhodanine-3-acetic acid. The strong electron-donating dithiafulvalene unit in these dyes broadens absorption, causes redshift, reduces aggregation, and promotes rapid regeneration of the oxidized dye in DSSCs, resulting in improved photovoltaic performance compared to a dye without an additional donating group (PCE = 3.96%).

Tian et al. [46] developed three novel dyes (**D14**–**D16**), with dye **D15** delivering the best photovoltaic performance (PCE = 7.21%, V_OC_ = 0.723 V, J_SC_ = 14.84 mA cm^−2^, FF = 0.672). The planarization of the side chain in the donor slightly increased HOMO levels, enhancing light-harvesting capabilities, though the effect on ICT processes was minimal.

Using a benzo[a]carbazole donor moiety with a cyanoacryl acid as its acceptor group, Qian and colleagues [47] investigated the effect of various electron-rich spacers such as thiophene (**D17**), furan (**D18**), and oligothiophene (**D19**) as π-linkers. Dye **D19** demonstrated a broad incident photon-to-current efficiency (IPCE) response with photocurrent signals extending up to approximately 740 nm, covering most of the UV-visible spectrum. Its absorption maximum was redshifted to 477 nm, with a molar extinction coefficient of 3.24 M^−1^ cm^−1^. This indicates that expanding the electron-rich conjugated π-linkers not only enhances donor-acceptor interactions but also reduces the energy gap of the organic dyes. **D19**-based dye achieved the best PV performance with a J_SC_ of 14.8 mA cm^−2^, V_OC_ of 0.744 V, and an FF of 0.68, resulting in a PCE of 7.54%.

Another study, which focused on the effect of various π-linkers on the performance of DSSCs, has also been reported [48]. These dyes (**D20**–**D23**) contain a carbazole donor and a triarylamine acceptor, as well as a cyanoacrylic acid-anchoring group. Of these, dye **D20** exhibited the highest PV performance with a PCE of 8.67%, notably outperforming dyes (**D21**–**D23**). The inclusion of the 9-hexyl-2-(hexyloxy)-9*H*-carbazole unit in triarylamine offered several advantages, including: (i) a redshift of the absorption peak, (ii) an increased maximum molar absorption coefficient, reduced charge recombination in cobalt and iodine cells, and enhanced photocurrent/photovoltage. Taken together, these features led to an improvement in the PCEs of these cells.

Work by Wu et al. [49] has focused on designing effective DSSCs by assessing various donors such as carbazole (**D24**), phenothiazine (PTZ), or triphenylamine (tPA), which were coupled to an oligothiophene π-spacer and a cyanoacrylic acid acceptor. All these dyes exhibited strong absorption around 400 nm due to their rod-like structures. The carbazole-based dye **D24** showed the highest PCE of 5.92%, surpassing tPA (5.30%) and PTZ (5.40%) due to its redshifted absorption band (442 nm) and superior electron-donating capacity. The highest V_OC_ value of 0.740 V was attributed to the larger dihedral angle between the donor and the π-bridge.

To design carbazole dendrons up to the fourth generation as donors, a bithiophene was used as π-linkage and cyanoacrylic acid was used as the acceptor (**D25**) [50]. Dye **D25**, containing a first-generation dendron (the smallest molecular volume), exhibited the highest PCE of 5.10% (J_SC_ = 9.89 mA cm^−2^, V_OC_ = 0.720 V, FF = 0.73). Increasing the size or generation of the carbazole dendritic donor enhanced light absorption but reduced dye uptake per unit TiO_2_ area due to the high molecular volume. Electrochemical impedance spectroscopy (EIS) revealed that the donor’s size or generation significantly affected charge-transfer resistance at the TiO_2_/electrolyte interface, impacting V_oc_ and, consequently, the PCE.

Three organic dyes based on indolo[3,2-b]carbazole as the donor moiety and various electron-withdrawing groups as acceptors were synthesized (**D25**–**D26**), and their performance in solar cell applications was examined [51]. The last dye, presented in the article, demonstrated the broadest light-response range (300–770 nm) in the incident monochromatic photoelectron conversion-efficiency curve, owing to its narrow bandgap. Among these three dyes, dye **D26** exhibited the best device performance with a PCE of 5.41% (J_SC_ of 12.55 mA cm^−2^, V_OC_ of 0.745 V, and FF of 0.59). The introduction of an alkylated indolo[3,2-b]carbazole moiety efficiently suppressed dye aggregation, resulting in better PCE for all three dyes.

Periyasamy et al. [52] introduced three new carbazole-based compounds (**D28**), featuring a carbazole donor, a 2-chlorophenothiazine π-bridge, and different acceptors, such as triethylamine, ethyl thiophene-2-glyoxylate, and 4-nitrophenol (**D28**). Among these, dye **D28** demonstrated outstanding photovoltaic properties with a PCE of 5.68%, attributed to its superior π-type transitions contributing to absorption in the visible region. In contrast, compounds with acceptors triethylamine and ethyl thiophene-2-glyoxylate showed lower efficiencies, with PCEs of 3.73% (V_OC_ = 0.714 V, J_SC_ = 7.08 mA cm^−2^, FF = 0.691) and 3.97% (V_OC_ = 0.720 V, J_SC_ = 8.37 mA cm^−2^, FF = 0.713), respectively.

Wagner et al. [53] reported carbazole-substituted dialkoxybenzodithiophene sensitizers (**D29**–**D30**), which included carbazole-based donors and cyanoacrylic acid acceptors separated by benzodithiophene with different alkoxy substituents. These dyes exhibited PCE values of 4.49%, 5.27% (**D29**), and 5.60% (**D30**). The extension of the π-system between carbazole and thiophene by a benzene ring achieved a 20 nm red shift, with the highest efficiency due to the long alkoxy chain that effectively reduced recombination by hindering oxidized electrolyte access to the film surface.

Researchers have explored the effect of co-sensitization by synthesizing two carbazole-based dyes (**D31**–**D32**) [54]. These dyes exhibited efficiencies of 6.0%, and 5.1%, respectively. The dye incorporating a dicarbazole motif donor and benzoic acid acceptor (**D31**) achieved the highest PCE, with this result being attributed to the existence of a more positive LUMO level, arising due to the nature of its conjugated structure. Dyes (**D31** and **D32,** which consist of an indolocarbazole donor, benzotriazole-based linker, and benzoic acid acceptor), showed improved efficiency (10.3%) in cosensitization due to the presence of additional fluorine atoms in the benzotriazole unit, which enhanced the light-capturing range of both dyes on the TiO_2_ film.

Kumaran et al. [55] synthesized three sensitizers that incorporate bromo phenyl carbazole as a donor, along with various acceptor moieties: acrylic acid (**D33**), cyanoacrylic acid (**D34**), and cyanoacetohydrazide acid (**D35**). These sensitizers are linked by a π-bridge consisting of a carbon atom. Among them, **D35** achieved the highest power-conversion efficiency of 8.12%, while D34 followed with 7.46%. D35 also demonstrated a significant redshift, with a *λ*_max_ of 481 nm, and exhibited greater electron delocalization throughout the molecule. The introduction of a phenyl moiety at the N-position of the carbazole resulted in a non-planar structure, which effectively prevents aggregation and enhances efficiency in dye-sensitized solar cells. Overall, these dye molecules show promising performance when utilized in DSSCs.

#### 3.1.2. D-D-π-A Architecture

**Figure 5:**
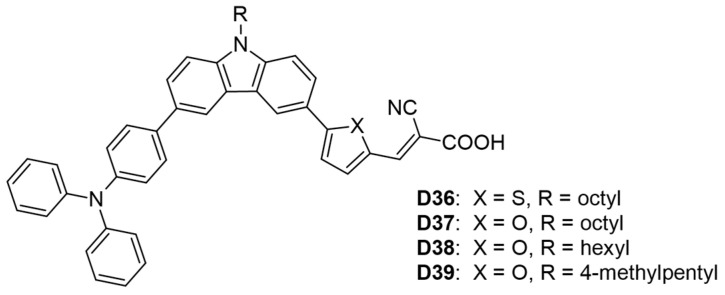
Structure of the carbazole-based molecules with D-D-π-A architecture (**D36** to **D39**) as sensitizers for DSSCs.

In another study, He and his colleagues [56] synthesized four carbazole-based dyes (**D36**–**D39**), incorporating linear or branched alkyl side chains as donors, with triphenylamine serving as an additional donor and cyanoacrylic acid as the acceptor (Table 2 and Figure 5). Dye **D37**, which included an octyl-substituted carbazole and a disubstituted furan, exhibited the highest photovoltaic performance, achieving a PCE of 6.68% (V_OC_ = 0.724 V, J_SC_ = 13.26 mA cm^−2^, FF = 0.66). In contrast, dye **D36**, featuring a thiophene linker, displayed a lower PCE of 5.59% and an absorption maximum of 442 nm, which has been attributed to the lower electronegativity of sulfur versus oxygen. Additionally, the linear alkyl side chain in dye (**D38**) resulted in a higher PCE than the branched side chain in dye (**D39**) (6.35% vs. 6.04%).

**Table 2:** Photovoltaic parameters of the DSSCs based on carbazole-based sensitizers with iodine electrolyte D-D-π-A architecture.

Dye No.	λ_max_ nm, (ε × 10^4^ M^−1^ cm^−1^)	PCE (%)	FF	V_OC_ (V)	J_SC_ (mA cm^−2^)
**D36**	442 (3.00)	5.59	0.678	0.741	11.12
**D37**	468 (3.67)	6.68	0.661	0.724	13.26
**D38**	468 (3.64)	6.35	0.666	0.737	12.31
**D39**	468 (3.35)	6.04	0.655	0.722	12.14

Note: A Pt electrode was utilized as counter electrode and I^−^/I_3_^−^ redox couple as its electrolyte. Absorption maxima in CH_2_Cl_2_ solution.

#### 3.1.3. D-(π-A)_2_ Architecture

**Figure 6:**
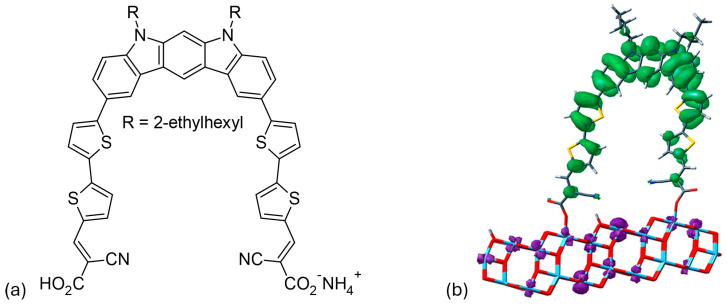
(**a**) Chemical structure of the **D40** dye. (**b**) Frontier molecular orbitals illustrate the HOMO-to-LUMO transition state of the **D40**/(TiO_2_)^70^ system, with green indicating holes and purple representing electrons. Adapted with permission from reference [57]. Copyright (2014), American Chemical Society.

Su and colleagues [57] developed an innovative synthesis method for indolo[2,3-b]carbazole by a double intramolecular Buchwald–Hartwig reaction. This method produces **D40** (Figure 6a), which has two π-bridged 2-cyanoacrylic acid groups that act as bidentate anchors. The dye uses an N-alkylated indolo[2,3-b]carbazole as a geometrically fixed core and reaches an absorption maximum at 467 nm. The effective electron transfer from the HOMO of the indolo[2,3-b]carbazole to the LUMO of a TiO_2_ nanocluster, in combination with the bidentate anchoring, leads to an energy-conversion efficiency of 6.02%. The device has a short-circuit current density of 12.45 mA/cm^2^, an open-circuit voltage of 0.69 V, and a fill factor of 0.70. As shown in Figure 6b, **D40** is connected to the (TiO_2_)_70_ nanocluster, with the distance between the two carboxyl groups being 15.2 Å. It was found that the lowest singlet excitation of **D40**/TiO_2_ involves only one HOMO→LUMO transition. The HOMO is primarily localized on the electron-rich indolo[2,3-b]carbazole backbone, while the LUMO is predominantly located on the semiconductor, as shown in the frontier molecule orbital representation in Figure 6b. This complete spatial separation of HOMO and LUMO, and the associated transition, facilitates the efficient electron transfer and injection of D40 into TiO_2_.

#### 3.1.4. D-π-D-A, D-(π-D-A)_2_, D-(π-D-A)_3_ Architecture

**Figure 7:**
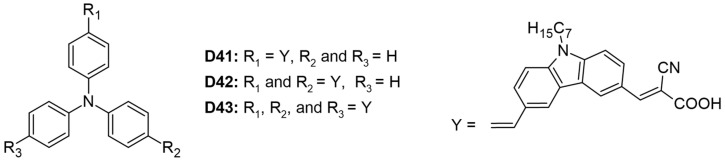
Structure of the carbazole-based molecules with D-π-D-A (**D41**), D-(π-D-A)_2_ (**D42**), and D-(π-D-A)_3_ (**D43**) architectures as sensitizers for DSSCs.

Three organic dyes (**D41**–**D43**) were synthesized, each featuring a triphenyl core donor, carbazole donors, and cyanoacrylic acid acceptors, as illustrated in Figure 7 [29]. Among these, dye **D43** demonstrated the highest values for V_OC_ and PCE at 5.44%, attributed to its greater number of anchoring groups. These groups enhanced electronic coupling with TiO_2_, thereby improving light-harvesting efficiency. However, the study did not specify whether all three anchoring moieties were simultaneously attached to the semiconductor. The molar extinction coefficients increased proportionally with the number of anchoring groups: 4.55, 6.50, and 7.50 for dyes **D41**, **D42**, and **D43**, respectively. Dye **D41** exhibited a redshifted absorption maximum at 423 nm due to its unidirectional charge-transfer process, whereas dye **D42** showed a more blueshifted absorption maximum at 397 nm (Table 3).

**Table 3:** Photovoltaic parameters of the DSSCs based on carbazole-based sensitizers with D-π-D-A, D-(π-D-A)_2_, and D-(π-D-A)_3_ architecture.

Dye No.	λ_max_ nm, (ε × 10^4^ M^–1^ cm^–1^)	PCE (%)	FF	V_OC_ (V)	J_SC_ (mA cm^–2^)
D41	423 (4.55)	5.31	0.682	0.731	10.65
D42	397 (6.50)	4.70	0.665	0.758	9.40
D43	402 (7.50)	5.44	0.572	0.770	12.41

Note: A Pt electrode was utilized as a counter electrode and the I^−^/I_3_^−^ redox couple as its electrolyte. Absorption maxima in DCM solution.

#### 3.1.5. D-A-π-A Architecture

**Figure 8:**
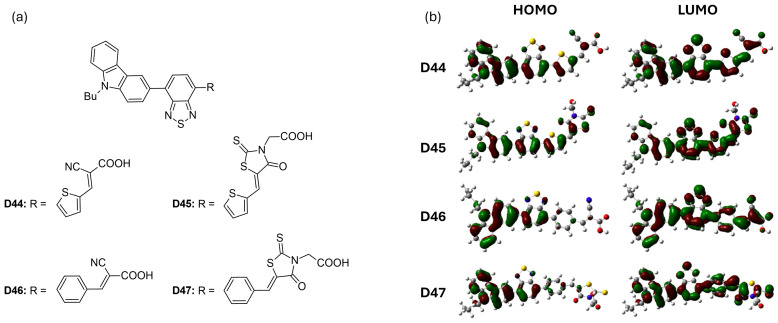
(**a**) Structure of the carbazole-based molecules with D-A-π-A architecture (**D44** to **D47**) as sensitizers for DSSCs. (**b**) Frontier molecular orbital of the dyes. Adapted with permission from reference [58]. Copyright (2014), Elsevier.

Han et al. [58] introduced four new D-A-π-A dyes (**D44** to **D47**), each featuring distinct π-bridges and acceptor groups. The structures of these dyes are illustrated in Figure 8a. Among these, dye **D46**, featuring a benzene bridge, achieved the highest power-conversion efficiency of 5.40% (V_OC_ = 0.710 V), outperforming dye **D44**, which had a thiophene bridge (PCE = 5.07%, V_OC_ = 0.610 V). When comparing the effects of different anchoring groups, dyes containing a cyanoacrylic acid moiety (**D44** and **D46**) exhibited superior photovoltaic performance compared to dyes **D45** and **D47**, which utilized rhodamine acetic acid (Table 4). This difference is attributed to the methylene-attached carboxylic group in rhodamine acetic acid, which hinders effective electron injection from the dyes into the conduction band edge of TiO_2_, as illustrated in Figure 8b. Additionally, Figure 8b shows that the LUMOs of all four dyes possess similar frontier molecular orbitals that extend throughout the molecules. However, the contributions of the π-bridges (benzene in **D46** and **D47** versus thiophene in **D44** and **D45**) vary in their delocalization. The thiophene π-bridges exhibit greater delocalization due to a smaller dihedral angle between the benzothiadiazole and thiophene rings. This variation influences both the absorption characteristics and charge-injection capabilities of the dyes.

**Table 4:** Photovoltaic parameters of the DSSCs based on carbazole-based sensitizers with D-A-π-A architecture.

Dye No.	*λ*_max_ nm, (ε × 10^4^ M^−1^ cm^−1^)	PCE (%)	FF	V_OC_ (V)	J_SC_ (mA cm^−2^)
**D44**	486 (3.06)	5.07	0.690	0.610	12.08
**D45**	507 (4.54)	1.65	0.710	0.560	4.12
**D46**	441 (2.17)	5.40	0.690	0.710	10.99
**D47**	446 (3.47)	3.81	0.870	0.630	6.98

Note: A Pt electrode was utilized as counter electrode and I^−^/I_3_^−^ redox couple as its electrolyte. Absorption maxima in 1:10 MeOH:CHCl_3_.

#### 3.1.6. A-π-D-π-A-π-A Architecture

**Figure 9:**
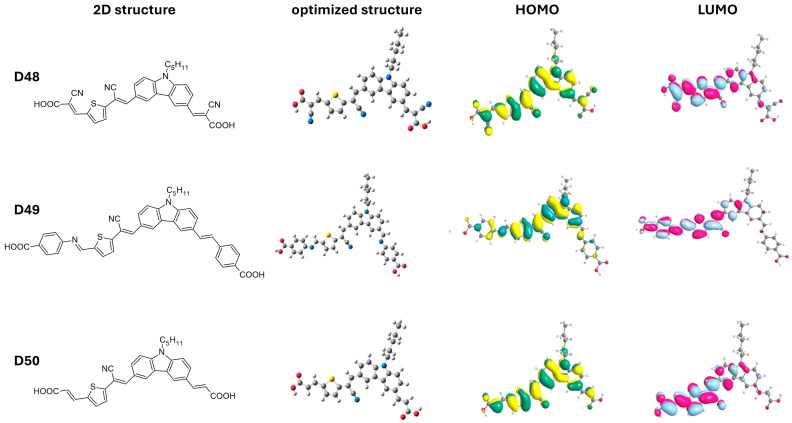
Two-dimensional structure, optimized geometry, and its corresponding HOMO and LUMO orbitals of dyes **D48** to **D50**. Adapted with permission from reference [59]. Copyright (2020), Elsevier.

Ghasempour Nesheli and colleagues [59] developed a series of di-anchoring carbazole-based dyes (**D48**–**D50**) with an A-π-D-π-A-π-A structure (Table 5 and Figure 9). The electron-rich carbazole moiety in these dyes was attached to various acceptor/anchoring groups (i.e., cyanoacetic acid (**D48**), 4-aminobenzoic acid (**D49**)**,** and malonic acid (**D50**)) and π-spacers (vinylene and cyano-vinyl thiophene). Dye **D49** exhibited the best photovoltaic performance with a PCE of 2.27%, J_SC_ of 5.95 mA cm^−2^, V_OC_ of 0.54 V, and FF of 71%. This enhanced performance is attributed to the strong electron-withdrawing properties of the 4-aminobenzoic acid acceptor. According to the theoretical findings, the HOMO levels for the dyes **D48** (−5.98 eV), **D49** (−5.68 eV), and **D50** (−5.72 eV) are sufficiently lower than the redox couple’s electrode potential (−5.2 eV) to promote regeneration. Additionally, as the obtained LUMO levels of **D48** (−3.40 eV), **D49** (−2.98 eV), and **D50** (−2.94 eV) are higher than the CB of TiO_2_ (−4.2 eV), it is evident that electron injection is also promoted. It is observed in Figure 9 that the other anchoring group connected to the vinylene unit does not participate in the electron-injection process.

**Table 5:** Photovoltaic parameters of the DSSCs based on carbazole-based sensitizers with A-π-D-π-A-π-A architecture.

Dye No.	λ_max_ nm, (ε × 10^4^ M^−1^ cm^−1^)	PCE (%)	FF	V_OC_ (V)	J_SC_ (mA cm^−2^)
**D48**	397 (5.25)	2.14	0.720	0.520	5.66
**D49**	426 (6.93)	2.27	0.710	0.540	5.95
**D50**	392 (4.53)	1.69	0.710	0.480	4.99

Note: A Pt electrode was utilized as a counter electrode and the I^−^/I_3_^−^ redox couple as its electrolyte. Absorption maxima in DMF solution.

**Figure 10:**
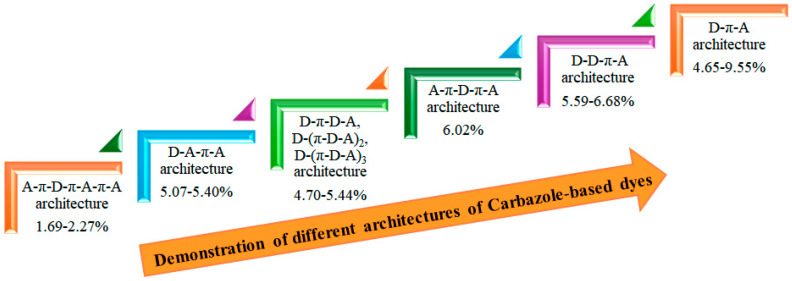
Enhancement PCE by different architectures.

We demonstrate the improvement in power-conversion efficiency achieved through various architectures of carbazole-based dyes, as illustrated in Figure 10. By utilizing a simple donor-π-acceptor structure within an I^−^/I_3_^−^ redox electrolyte solution, achieved a promising PCE of 9.55% [39]. This impressive performance underscores the effectiveness of the D-π-A motif in enhancing the light-harvesting capabilities of organic sensitizers. The D-π-A architecture is particularly beneficial because it promotes efficient charge transfer and boosts light absorption. The electron-donating carbazole units in these dyes provide a robust absorption spectrum, while the π-conjugated linkers enhance electronic delocalization, resulting in improved photovoltaic performance.

Moreover, incorporating various substituents on the carbazole core allows for the fine-tuning of optical and electronic properties, making them suitable for specific applications. These findings strongly indicate that the D-π-A motif is a promising approach for developing efficient pure organic sensitizers. Future research should focus on exploring different structural modifications and combinations to further enhance both PCE and stability under operational conditions. By systematically optimizing these parameters, we can advance the potential of carbazole-based dyes in the field of organic photovoltaics, paving the way for more sustainable and cost-effective energy solutions.

**Figure 11:**
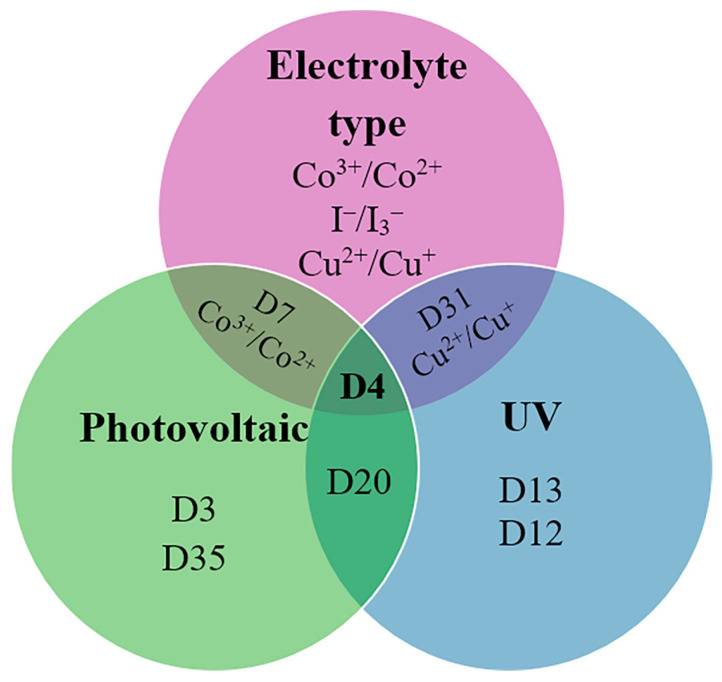
Euler diagram of the carbazole-based sensitizers.

Figure 11 presents a Euler diagram that serves as a tool for identifying dye molecules with optimal photovoltaic performance, particularly focusing on their absorption characteristics in the near-infrared (NIR) region and their redox properties. Among the various dyes analyzed, the **D4** dye stands out with a power-conversion efficiency of 9.55%, representing a remarkable 32% improvement over the widely studied N719 dye [39]. This significant advancement underscores the potential of **D4** as a more effective option for enhancing the efficiency of solar cells. The superior performance of the **D4** dye can be attributed to several key factors. Firstly, its exceptional electron-injection efficiency allows for a more effective transfer of electrons from the dye to the semiconductor, which is crucial for maximizing current output. This increased efficiency is complemented by the dye’s ability to suppress sensitizer aggregation, a common issue that can lead to reduced performance by blocking effective light absorption and hindering charge transport. Additionally, **D4** demonstrates a remarkable capability to minimize interface charge recombination, which is a significant loss mechanism in solar cells. By effectively reducing this recombination, the dye not only preserves the generated charge but also enhances the overall charge-carrier lifetime. This increased electron lifetime is vital for improving the stability and performance of the solar cell, allowing for more prolonged energy conversion before the charges recombine and dissipate. Overall, the findings highlight the importance of material properties in the design of efficient dye-sensitized solar cells. The advancements represented by the **D4** dye suggest that further exploration of similar materials could lead to even greater improvements in PV performance. Future research should focus on optimizing the molecular structure and exploring other dye combinations that can maximize light absorption and charge transport, ultimately contributing to the development of more efficient and commercially viable solar energy solutions.

To summarize, this section covers a wide range of carbazole derivatives, each divided into subsections based on their structural variations. Researchers from several teams have improved carbazole as a donor material in devices that convert light energy into electrical energy. The effects of different positions of carbazole derivatives on their properties were analyzed. It was found that the 2,7-carbazole configuration exhibited better planar conjugation and higher potential. The results emphasize the significant potential of carbazole derivatives for future use in DSSCs as they have inherent advantages. Studies have shown that modifying the structure of carbazole dyes can significantly increase their efficiency in DSSCs by achieving high carrier mobilities. With the ongoing advances in molecular engineering and the development of panchromatic dye designs, carbazole-based materials will perform even better in the future.

#### 3.1.7. Comparison of Carbazole with Different Donor Materials

Carbazole is characterized in organic electronics by its significant influence on the energy levels of the frontier molecular orbitals and molecular planarity, especially in comparison to other donor materials such as thiophene and benzene. The nitrogen atom in the ring structure of carbazole enhances its ability to donate electrons, leading to higher energy levels of frontier molecular orbitals. In contrast, thiophene derivatives generally exhibit lower energy levels of frontier orbitals, which is influenced by the presence of sulfur that affects the electronic distribution [60,61]. While thiophene-based materials can improve charge-transport properties, their energy levels do not always match those of carbazole derivatives under certain configurations. Benzene, which donates fewer electrons than carbazole and thiophene, typically has even lower energy levels of frontier orbitals [38]. The absence of heteroatoms in benzene reduces its reactivity and flexibility in tuning electronic properties, limiting its suitability for applications requiring high energy levels [61].

In terms of molecular planarity, carbazole’s structure supports significant planarity, especially with optimized side chains that minimize dihedral angles, thereby enhancing charge-transport efficiency [46,62]. However, certain substitutions can introduce non-planarity due to steric interactions, potentially impacting electronic properties negatively [38]. Thiophene derivatives vary in planarity depending on their design but generally maintain a beneficially planar configuration for effective π-π stacking interactions crucial to charge transport [60]. The inclusion of alkyl chains can influence thiophene derivatives’ planarity, with longer chains potentially reducing it due to increased steric hindrance [60,63].

Benzene’s inherent fully planar structure contrasts with its limited versatility in modifying electronic properties through structural changes, restricting its effectiveness as a donor material compared to carbazole and thiophene derivatives [38,61]. Despite its planarity, benzene lacks the necessary electronic properties to function effectively in hole-transport applications. Carbazole’s advantageous balance of energy levels and molecular geometry continues to position it favorably in numerous organic electronic applications.

## 4. Carbazole-Based Hole-Transporting Materials for Solid-State DSSCs

Compared to inorganic hole-transporting materials, organic HTMs offer numerous advantages, including superior thin-film formation, cost-effectiveness, excellent solubility, the possibility of a wide variety of compositions, environmental friendliness, solution processability, mechanical flexibility, tunable electronic properties, and ease of fabrication. Most HTMs are synthesized by way of metal-catalyzed cross-couplings, such as the Stille, Suzuki, Buchwald–Hartwig, and Ullmann reactions. The glass-transition temperature (*T*_g_) is a critical property for HTMs, as it represents the reversible transition in amorphous materials, allowing molecules to move rapidly when heated. The molecular glass properties of HTMs are essential for effectively filling the pores in TiO_2_, enhancing the contact area between TiO_2_ and the HTM.

While liquid electrolytes can be used in dye-sensitized solar cells to achieve higher power conversion efficiencies, they also have drawbacks like toxicity, leakage, and volatilization [64]. Consequently, the development of solid-state electrolytes has become increasingly important in DSSC research.

**Figure 12:**
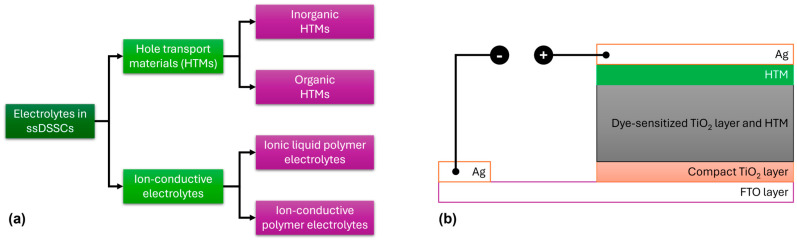
(**a**) Classification of solid-state electrolytes for ssDSSC applications. (**b**) Schematic representation of the commonly used device architecture used in ssDSSCs.

This section of the review focuses on solid-state electrolytes based on carbazole for solid-state DSSC (ssDSSC) applications. HTMs can be broadly classified into inorganic and organic types, while ion-conductive electrolytes are divided into ionic liquid polymer electrolytes and ion-conductive polymer electrolytes (Figure 12a). A reliable HTM for ssDSSCs must meet several criteria: (1) It should have an energy level that matches the dye, (2) it should be processed gently to avoid damaging the photoanode, (3) it should exhibit high hole mobility, and (4) it should demonstrate good photoelectrochemical stability [65,66,67,68,69,70].

**Figure 13:**
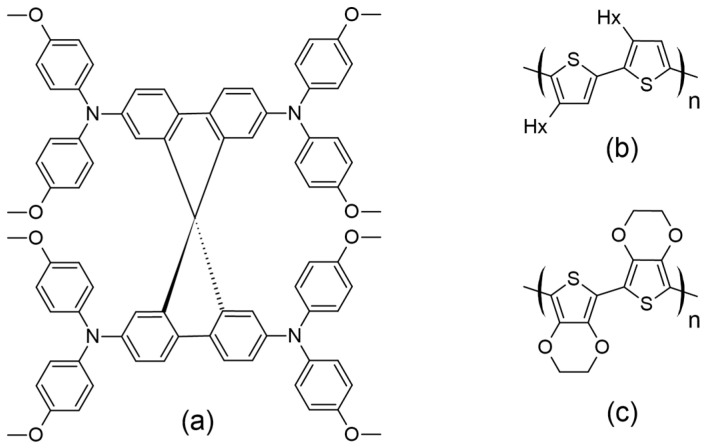
Structures for (**a**) spiro-OMeTAD, (**b**) P3HT, and (**c**) PEDOT as commonly used HTMs in ssDSSCs.

A major difference between DSSCs consisting of a liquid electrolyte and those containing a solid-state electrolyte is the nature of the HTM, while, additionally, in the latter, the cathode electrode is commonly constructed using Ag (Figure 12b). Several organic HTMs, such as 2,2′,7,7′-tetrakis-(*N*,*N*-di-4-methoxyphenylamino)-9,9′-spirobifluorene (spiro-OMeTAD), poly(3-hexylthiophene) (P3HT), and poly(3,4-ethylenedioxythiophene) (PEDOT), have been extensively studied in ssDSSCs (Figure 13). For organic HTMs, two key factors influencing ssDSSC performance are electrolyte conductivity and electrolyte filling within the TiO_2_ film.

### 4.1. Dopants for Hole-Transport Materials

**Figure 14:**
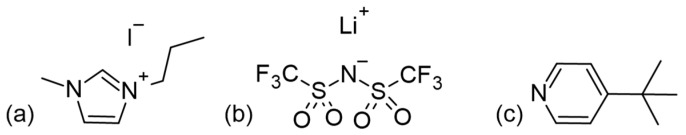
Structures for (**a**) MPII, (**b**) LiTFSI, and (**c**) tBP as commonly used additives in ssDSSCs.

In the context of HTMs, there is often confusion in the literature between additives and proper dopants (p-dopants). Additives, like 1-methyl-3-propylimidazolium iodide (MPII), lithium bis(trifluoromethanesulfonyl)imide (LiTFSI), and 4-*tert*-butylpyridine (tBP) (the structures of which are presented in Figure 14) are dissolved in the HTM precursor solution to be applied after dye sensitization. They do not directly affect the HTM itself but may migrate to the TiO_2_ surface to adjust energy levels, passivate exposed surfaces, improve charge injection, and reduce charge recombination at the TiO_2_–HTM interface [71,72,73,74,75,76,77,78,79]. Proper dopants, on the other hand, directly affect solid-state hole conductors by partially oxidizing the HTM material. This oxidation creates vacancies in the solid-state film, significantly increasing hole mobility and, ultimately, conductivity [79]. Dopants with a more positive redox potential than the HTM (or strong Lewis acids) that act as *p*-type dopants promote electron removal from the hole conductor. Most of the dopants reviewed have been applied to the spiro-OMeTAD molecule, but they can also enhance conductivity in other hole transport materials [80,81,82].

### 4.2. Spiro-OMeTAD

Spiro-OMeTAD, the most commonly used HTM, was first synthesized by the Salbeck group in 1997 [83]. The concept behind spiro-OMeTAD involves using an *sp*^3^ hybridized atom to link two or more π-conjugated systems while maintaining their electrical properties. The cross-shaped molecular structure of spiro-linked HTMs (Figure 13a) offers benefits such as rigidity, high stability, and efficient excimer formation [84]. Its design minimizes intermolecular bonding interactions, resulting in a lower *T*_g_ (125 °C). Heating the material above *T*_g_ to the melting temperature (248 °C) ensures full coverage of the film, optimizing hole transmission [85]. The presence of methoxy groups at the *para* position to nitrogen (known as an electron-rich atom) and their mesomeric electron-donating effect led to a low oxidation potential (i.e., a relatively high HOMO level). It has been shown that by moving the methoxy groups to other sites, the HOMO level is stabilized to around 0.12 eV. Since then, more information has come to light about the modification of spiro-linked semiconductors to improve their electrical and chemical properties [84]. A ssDSSC made with spiro-OMeTAD was first reported by Grätzel and colleagues in 1998 [86], but this was associated with a relatively meager efficiency of just 0.74% (using N(PhBr)_3_SbCl_6_ as a dopant and Li[(CF_3_SO_3_)_2_] as an additive). Due to the complexities associated with synthesizing spiro-OMeTAD, which also renders its synthesis economically costly, researchers explored other HTMs that offer either comparable or superior hole mobility but are vastly easier to synthesize, and with a reduced production cost.

### 4.3. Advancements in Carbazole-Based HTMs

**Figure 15:**
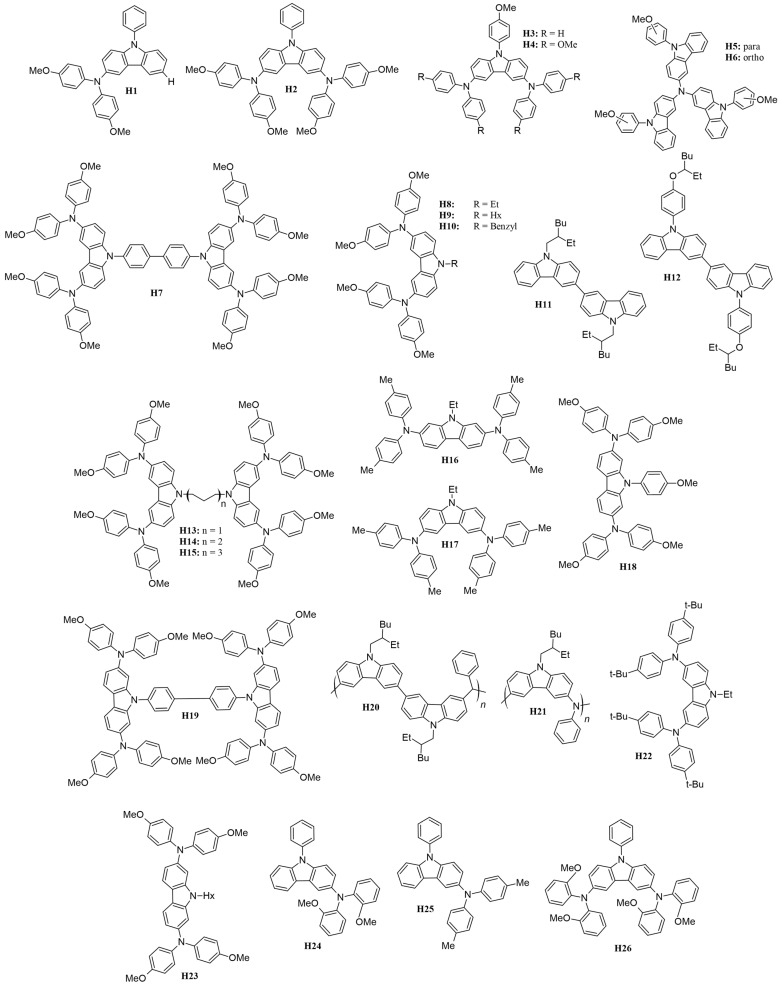
Structures of carbazole-based HTMs used in ssDSSCs.

Several carbazole-based HTMs with different molecular structures, stability, optical, and electrochemical characteristics have been studied, offering insights into the field’s progress, are presented in Figure 15 with various moieties and attached to either 3,6- or 2,7-positions.

**Figure 16:**
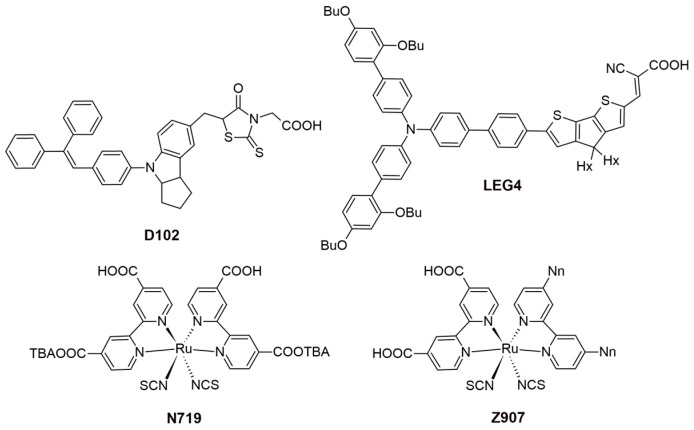
Structures of the sensitizers used in ssDSSCs.

Recent studies have highlighted the effects of methoxy group substitutions at the *para*-position of diphenylamine in carbazole-based hole-transport materials. Researchers have designed and synthesized four such carbazole-based HTMs, labeled **H1** to **H4** [87,88]. These materials, developed as effective molecular glasses, are suitable for use in solid-state dye-sensitized solar cells. The performance of these HTMs was influenced by mono- or di-substitution of dimethoxy-diphenylamine groups at the 3 and/or 6 positions of the carbazole moiety. By utilizing a 3,6-substituted carbazole molecular glass derivative **H2**, the researchers achieved a PCE of 3.4% and a fill factor of 0.53. This performance is comparable to that of a reference device using spiro-OMeTAD, which has a PCE of 3.5% [87]. Additionally, further studies on HTMs suggest that incorporating methoxy groups into diphenylamine moieties can enhance photovoltaic-conversion efficiency. For instance, devices equipped with HTMs featuring methoxy groups **H4** have achieved PCEs of 1.75%, even without further optimization [88]. A comprehensive characterization of the HTM properties, such as optical, thermal, electrochromic, and photovoltaic parameters, are presented in Table S1. In this research, they utilized an indoline-based sensitizer (**D102**), whose structure is presented in Figure 16.

To expand the range of inexpensive low-molecular-weight amorphous compounds for ssDSSCs, the researchers synthesized star-shaped carbazole derivatives with a nitrogen core in which the methoxy groups are positioned differently on the *N*-phenyl moiety [89]. These derivatives are named **H5** and **H6**, corresponding to the *para* and *ortho* position of the methoxy group, respectively (Figure 14). The *ortho* position in **H6** causes the phenyl moiety to be more inclined, possibly affecting its packing configuration and solubility (for example, **H6** is less soluble when compared to **H5**). Despite these differences, both HTMs exhibit very similar optical and electronic properties. Their band gaps are 2.76 eV, which is sufficiently low to avoid interference with the absorption, photoexcitation, and charge transfer of the **D102**. The solid-state ionization potential (IPs) of HTMS agrees well with that of **D102** and provides a driving force of more than 0.2 eV for efficient charge transfer and regeneration of the photooxidized dye. Under simulated AM 1.5 irradiation, **H5** achieves a higher J_sc_ of 8.85 mA cm^−2^, resulting in a PCE of 2.23%. In contrast, **H6** shows a significantly lower J_sc_ of 1.57 mA cm^−2^, which is probably due to insufficient pore filling, poor interface quality, or poor charge-transport properties.

To design a symmetric carbazole with a structure similar to spiro-OMeTAD, Xu and colleagues synthesized mono-carbazole (**H4**) and bis-carbazole-based (**H7**) hole-transport materials, as illustrated in Figure 14 [90]. The large conjugated system in **H7** facilitates more efficient π-π stacking, resulting in higher conductivity and hole mobility compared to the monomeric system **H4**. This improved performance translates to better photovoltaic parameters, with **H7** achieving a notably high open-circuit voltage of 0.92 V, compared to 0.75 V for **H4**, owing to its slower recombination rate. Among the hole-transport materials reviewed, **H7** not only boasts the highest V_OC_ but also ranks second in terms of overall efficiency.

Researchers have examined how the length and bulkiness of the moiety attached to the nitrogen of the carbazole affect its properties. Specifically, they studied the effects of ethyl (**H8**), hexyl (**H9**), and benzyl (**H10**) groups [91]. They found that increasing the alkyl group from ethyl to hexyl lowers the glass-transition temperature (*T*_g_) and enhances hole mobility. This improvement is attributed to the increased flexibility of the hole-transport material as the alkyl chain lengthens. The hexyl chain introduces more free volume and reduces the rigidity of the HTM, which leads to better molecular packing and ordering. This enhanced packing improves π-π stacking interactions, which are essential for efficient charge transport and allow holes to move more freely through the material. In contrast, the benzyl moiety **H10** results in very low hole mobility due to poorer molecular packing in the solid state.

The purity of the HTM is a crucial factor, as demonstrated by Degbia and colleagues [92]. They employed a sublimation strategy to enhance the purity of **H8**, which led to a notable improvement in the solar cells’ power-conversion efficiency, rising from 0.82% to 1.62%.

A similar strategy was used to enhance carbazole-based dyes by examining the impact of substituents attached to the nitrogen atom of the carbazole structure. This study employed a bis(carbazole) configuration rather than the previously used mono(carbazole). Specifically, they incorporated 2-ethylhexyl (**H11**) and 4-[(2-ethylhexyl)oxy]phenyl (**H12**) as substituents [93]. The introduction of the oxy group in the **H12** moiety significantly lowered the HOMO level, thereby widening the band gap by approximately 0.11 eV compared to **H11**. This reduction in the HOMO level likely contributes to a slight decrease in V_OC_ for **H12**, as it may absorb photons within a shorter wavelength range and produce a lower voltage. Despite this, the 4-[(2-ethylhexyl)oxy]phenyl group resulted in a higher J_SC_ and overall PCE. This improvement is likely attributed to a more efficient charge-transport mechanism in **H12**, which enhances the extraction of charge carriers and generates more current.

Benhattab et al. [94] synthesized a series of carbazole-based twin molecules, **H13** to **H15**, which exhibit molecular glass behavior. By varying the length of the linker, it is possible to adjust the glass-transition temperature. Notably, reducing the chain length significantly enhances hole mobility, with improvements approaching an order of magnitude. Among the synthesized molecules, **H13**, which has the shortest chain length, achieved a promising power-conversion efficiency of 2.21%. The study found that the linker length influences both the molecules’ hole mobility and glass-transition temperature, with shorter linkers being associated with increased hole mobility.

Researchers also investigated the performance of 2,7- and 3,6-disubstituted carbazoles, identified in Figure 15 as **H16** and **H17**, respectively [95]. It was found that the 3,6-disubstituted carbazole **H17** demonstrated superior pore filling on the mesoporous TiO_2_ surface due to its increased solubility. This enhanced solubility led to better photovoltaic performance compared to the 2,7-disubstituted carbazole. Although the 2,7-disubstituted carbazole exhibited good driving force and hole mobility, its effectiveness was limited by its poorer solubility.

To enhance solubility, carbazole-based hole-transport materials were modified by substituting methoxy groups at the 2,7-positions, and the study was extended to include a di-carbazole configuration, labeled HTMs **H18** and **H19**, respectively [96]. For comparison, the performance of these materials was evaluated against HTMs with substitutions at the 3,6-positions, specifically HTMs **H4** and **H7** [90]. Consistent with previous research, the 2,7-positions yielded significantly better hole mobilities, nearly twice as high as those observed with the 3,6-substituted counterparts. Enhanced hole mobility led to improved photovoltaic performance for the carbazole-based HTMs. Specifically, the di-carbazole HTM **H19**, which has the highest PCE among the carbazole-based HTMs discussed in this review, achieved a PCE of 6.8%. In comparison, the mono-carbazole HTM **H18** exhibited a PCE of 5.8%. Both values surpass the performance of their 3,6-substituted analogs.

Polymeric carbazoles, specifically **H20** and **H21** (Figure 14), were also studied as potential hole-transport materials for solid-state dye-sensitized solar cells. In polymeric types, molecular weight (MW) plays a crucial role in conversion efficiency. The higher MW of **H20**, a bis(carbazole)-based polymer, resulted in a 38% increase in conversion efficiency compared to other HTMs reviewed. This improvement is attributed to its superior conductivity and highest hole mobility among the HTMs considered, which enhanced the fill factor, open-circuit voltage, and short-circuit current [97]. In contrast, **H21** (i.e., poly[*N*-(2-ethylhexyl)-3,6-carbazole-*alt*-aniline]), has significantly lower hole mobility than its bis(carbazole) counterpart and, as a result, achieved a power-conversion efficiency of only 1.5% [98]. Thus, from this observation, a bis(carbazole) configuration is beneficial for polymeric-type HTMs.

Research by Ryu et al. demonstrates that incorporating magnesium(II) bis(trifluoromethanesulfonyl)imide (Mg(TFSI)_2_) as a dopant in the hole-transporting tercarbazole compound (**H22**) can significantly enhance the energy-conversion efficiency of solid-state dye-sensitized solar cells, outperforming the traditional dopant, LiTFSI [99]. The ssDSSC featuring Mg(TFSI)_2_ achieved a power-conversion efficiency of more than 70% higher than that of the reference cell. This notable improvement is attributed to superior electron injection and hole-collection efficiencies, leading to a higher fill factor and a significantly increased short-circuit current due to reduced series resistance. These findings underscore the importance of selecting the right dopant for hole-transporting materials to optimize photovoltaic performance in ssDSSCs. The use of Mg(TFSI)_2_ shows considerable promise for enhancing device efficiency and overall performance in solar energy-conversion applications.

Leijtens and colleagues synthesized **H23**, which features a low glass-transition temperature, low melting point, and high solubility, thereby making it an attractive choice for use in solid-state dye-sensitized solar cells [100]. This compound outperforms its 3,6-substituted counterpart, namely **H9** [91], achieving a higher PCE of 2.94% compared to 1.81% for **H9**. Additionally, the researchers investigated an alternative dopant, tris(4-bromophenyl)aminium hexachloroantimonate (TBPA), as opposed to the commonly used tBP and LiTFSI. TBPA provided higher conductivity, with a value of 2 × 10^−5^ S cm^−1^, compared to 7 × 10^−7^ S cm^−1^ for the tBP/LiTFSI mixture. Consequently, devices using TBPA demonstrated improved efficiency, reaching 2.30% compared to 0.50% for devices with tBP and LiTFSI. This group also tested ssDSSCs with a thicker cell (6 µm) due to the increased solubility of the hole-transport material when paired with the new dopant. The performance of the thicker cell was comparable to that of 2 µm-thick cells, with no observed difference in open-circuit voltage in either cell thickness. This indicates that there was no recombination-induced voltage drop in the titania electron quasi-Fermi level.

Tomkeviciene et al. [101] synthesized the compounds **H24**, **H25**, and **H26** (Figure 15), which exhibit a relatively high degree of thermal stability and are capable of absorbing electromagnetic radiation in the 225–425 nm range. These compounds have ionization energies ranging from 5.04 to 5.56 eV. In their amorphous layers, they display hole-drift mobilities as high as 10^−3^ cm^2^/V·s and achieve a power-conversion efficiency of up to 0.54%. The findings suggest that the thermal, optical, and electrochemical properties of these compounds are significantly affected by the positioning of the methoxy groups.

In summary, carbazole-based HTMs have shown significant potential for improving the efficiency of dye-sensitized solar cells. Ongoing research continues to explore new molecular designs, synthesis methods, and dopants to further enhance the performance and stability of HTMs in ssDSSCs and other solar energy applications.

**Figure 17:**
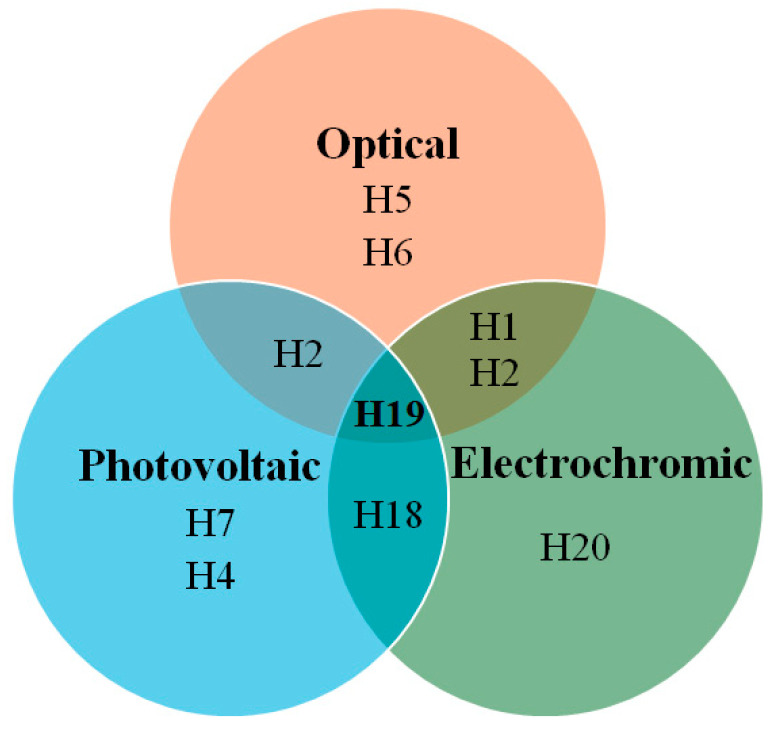
Euler diagram for HTMs.

In Figure 17, the Euler diagram illustrates that **H19** demonstrates excellent photovoltaic, electrochromic, and optical properties, with an absorption-emission peak at 416 nm. The synthesis of H19 is relatively straightforward, requiring only four steps and utilizing inexpensive starting materials. Its hole mobility is measured at 2.88 × 10⁻⁴ cm^2^/V·s, while the conductivity is 2.90 × 10⁻⁴ S/cm, which increases to 8.02 × 10⁻⁴ S/cm after light soaking. Theoretical calculations indicate that nitrogen atoms in the molecular units of **H19**, particularly those substituted at the 2,7-position, are more susceptible to oxidation by the p-type dopant, leading to the formation of radical cation intermediates. These radical cations exhibit varying optical properties due to their different linking topologies, aligning well with the UV-Vis absorption results observed post-doping. The **H19** solid-state dye-sensitized solar cell devices achieve a power-conversion efficiency of 6.8%, with a J_SC_ of 10.30 mA/cm^2^, V_OC_ of 0.88 V, and FF of 0.75.

## 5. Conclusions and Future Outlook

This review addresses various carbazole-based D-π-A dyes, focusing on factors that are crucial for solar cell efficiency, including the bulkiness of dye components, electron delocalization towards the anchoring group, energy levels, dark current, sufficient absorption in the near-infrared region, stability, and cell lifetime. In analyzing DSSCs based on different dyes, key findings have been established regarding the enhancement of photovoltaic performance in solar cells.

First, introducing alkyl and alkoxy groups into the donor or π segments of the dye helps reduce aggregation and decrease electrolyte interaction with the semiconductor oxide film, thereby improving cell stability. Second, cyano-acrylic acid has proven to be the most effective anchoring group, aligning well with the film surface. Third, expanding π conjugation broadens the absorption band, optimizing the short-circuit current. Moreover, the D-π-A alignment of donor and acceptor maximizes the intramolecular charge transfer, enhancing the sensitizer’s efficiency. Additionally, *para*-positioning of the donor group leads to higher dye loading. Lastly, co-sensitization with two dyes improves electron injection from light-excited dyes to TiO_2_ electrodes, resulting in longer cell lifetimes and a broader absorption range.

However, further research is needed in developing sensitizers and enhancing the performance of counter-electrodes and electrolytes. For instance, introducing multiple anchoring groups has been found beneficial for strong binding on semiconductor surfaces, effective electron injection, and greater device stability. By employing dyes that are strategically developed, it may be possible to create more affordable, stable, and efficient DSSCs.

This review also summarizes the relationship between molecular structure and electronic properties of different types of carbazole-based HTMs with versatile designs. Despite the extensive research on organic HTM derivatives, there remains an urgent need for materials with exceptional characteristics. The primary drawbacks of the state-of-the-art spiro-OMeTAD include its complex multistep synthesis, high cost, and limited stability, motivating material scientists and chemical engineers to design new materials with fewer synthetic steps.

In recent decades, a wide range of HTMs has been engineered, with a focus on better understanding the relationship between HTM structures and device performance. There is a continuing need to design novel HTMs or combine two HTMs with complementary properties to meet the requirements for achieving maximum efficiency in various energy applications. Continued research in this field is essential for gaining a deeper understanding of how the nature, chemical structure, and properties of HTMs influence the efficiency of electronic devices.

## Data Availability

The original contributions presented in the study are included in the article/Supplementary Material. Further inquiries can be directed to the corresponding authors.

## Supplementary Materials

The following supporting information can be downloaded at: https://www.mdpi.com/article/10.3390/molecules29215035/s1, Table S1: A comprehensive characterization of the optical, thermal, electrochromic, and photovoltaic parameters of the carbazole-based hole-transport materials.

## References

[1] Devadiga D., Selvakumar M., Shetty P., Santosh M., Chandrabose R.S., Karazhanov S. (2021). Recent developments in metal-free organic sensitizers derived from carbazole, triphenylamine, and phenothiazine for dye-sensitized solar cells. Int. J. Energy Res..

[2] Kumar D., Wong K.-T. (2017). Organic dianchor dyes for dye-sensitized solar cells. Mater. Today Energy.

[3] Murakami T.N., Koumura N. (2018). Development of next-generation organic-based solar cells: Studies on dye-sensitized and perovskite solar cells. Adv. Energy Mater..

[4] Konidena R.K., Thomas K.R.J., Park J.W. (2022). Recent advances in the design of multi-substituted carbazoles for optoelectronics: Synthesis and structure-property outlook. ChemPhotoChem.

[5] Han P., Zhang Y. (2024). Recent advances in carbazole-based self-assembled monolayer for solution-processed optoelectronic devices. Adv. Mater..

[6] Bekkar F., Bettahar F., Moreno I., Meghabar R., Hamadouche M., Hernáez E., Vilas-Vilela J.L., Ruiz-Rubio L. (2020). Polycarbazole and Its Derivatives: Synthesis and Applications. A Review of the Last 10 Years. Polymers.

[7] Karon K., Lapkowski M. (2015). Carbazole electrochemistry: A short review. J. Solid State Electrochem..

[8] Krucaite G., Grigalevicius S. (2021). 2,7(3,6)-diaryl(arylamino)-substituted carbazoles as components of oleds: A review of the last decade. Materials.

[9] Sathiyan G., Sivakumar E.K.T., Ganesamoorthy R., Thangamuthu R., Sakthivel P. (2016). Review of carbazole based conjugated molecules for highly efficient organic solar cell application. Tetrahedron Lett..

[10] Chetri R., Devadiga D., Ahipa T.N. (2024). A review on 4,4′-Dimethoxydiphenylamines bearing carbazoles as hole transporting materials for highly efficient perovskite solar cell. Sol. Energy.

[11] Sambathkumar S., Shakila P.B., Sundaram S., Subramaniam V., Raffaelle R.P., Nazeeruddin M.K., Morales-Acevedo A., Navarro M.B., Hepp A.F. (2024). Chapter Six—Organic hole-transporting materials for perovskite solar cells: Progress and prospects. Photovoltaics Beyond Silicon.

[12] Radhakrishna K., Manjunath S.B., Devadiga D., Chetri R., Nagaraja A.T. (2023). Review on Carbazole-Based Hole Transporting Materials for Perovskite Solar Cell. ACS Appl. Energy Mater..

[13] Zhao Y., Ma F., Qu Z., Yu S., Shen T., Deng H.X., Chu X., Peng X., Yuan Y., Zhang X. (2022). Inactive (PbI_2_)_2_RbCl stabilizes perovskite films for efficient solar cells. Science.

[14] Yang S., Liu Y., Lian X., Chu J. (2022). Inactive impurity stabilizes the highly efficient perovskite photovoltaics. Joule.

[15] NREL Best Research-Cell Efficiency Chart. https://www.nrel.gov/pv/interactive-cell-efficiency.html.

[16] O'Regan B., Grätzel M. (1991). A low-cost, high-efficiency solar cell based on dye-sensitized colloidal TiO_2_ films. Nature.

[17] Zhang L., Yang X., Wang W., Gurzadyan G.G., Li J., Li X., An J., Yu Z., Wang H., Cai B. (2019). 13.6% Efficient organic dye-sensitized solar cells by minimizing energy losses of the excited state. ACS Energy Lett..

[18] Ren Y., Zhang D., Suo J., Cao Y., Eickemeyer F.T., Vlachopoulos N., Zakeeruddin S.M., Hagfeldt A., Grätzel M. (2023). Hydroxamic acid pre-adsorption raises the efficiency of cosensitized solar cells. Nature.

[19] Michaels H., Rinderle M., Freitag R., Benesperi I., Edvinsson T., Socher R., Gagliardi A., Freitag M. (2020). Dye-sensitized solar cells under ambient light powering machine learning: Towards autonomous smart sensors for the internet of things. Chem. Sci..

[20] Ho J.K.W., Yin H., So S.K. (2020). From 33% to 57%—An elevated potential of efficiency limit for indoor photovoltaics. J. Mater. Chem. A.

[21] Shoaee S., Stolterfoht M., Neher D. (2018). The role of mobility on charge generation, recombination, and extraction in polymer-based solar cells. Adv. Energy Mater..

[22] Grätzel M. (2005). Dye-Sensitized Solid-State Heterojunction Solar Cells. MRS Bull..

[23] Brédas J.-L., Norton J.E., Cornil J., Coropceanu V. (2009). Molecular understanding of organic solar cells: The challenges. Acc. Chem. Res..

[24] Maldon B., Thamwattana N. (2020). Review of diffusion models for charge-carrier densities in dye-sensitized solar cells. J. Phys. Commun..

[25] Korir B.K., Kibet J.K., Ngari S.M. (2024). A review on the current status of dye-sensitized solar cells: Toward sustainable energy. Energy Sci. Eng..

[26] Koumura N., Wang Z.-S., Miyashita M., Uemura Y., Sekiguchi H., Cui Y., Mori A., Mori S., Hara K. (2009). Substituted carbazole dyes for efficient molecular photovoltaics: Long electron lifetime and high open circuit voltage performance. J. Mater. Chem..

[27] Periyasamy K., Sakthivel P., Venkatesh G., Vennila P., Sheena Mary Y. (2024). Synthesis and design of carbazole-based organic sensitizers for DSSCs applications: Experimental and theoretical approaches. Chem. Pap..

[28] Chandra Sil M., Chen L.-S., Lai C.-W., Lee Y.-H., Chang C.-C., Chen C.-M. (2020). Enhancement of power conversion efficiency of dye-sensitized solar cells for indoor applications by using a highly responsive organic dye and tailoring the thickness of photoactive layer. J. Power Sources.

[29] Elsherbiny D., Yildirim E., El-Essawy F., Abdel-Megied A., El-Shafei A. (2017). Structure-property relationships: Influence of number of anchoring groups in triphenylamine-carbazole motifs on light harvesting and photovoltaic performance for dye-sensitized solar cells. Dye. Pigment..

[30] Kakiage K., Aoyama Y., Yano T., Otsuka T., Kyomen T., Unno M., Hanaya M. (2014). An achievement of over 12 percent efficiency in an organic dye-sensitized solar cell. Chem. Commun..

[31] Kakiage K., Aoyama Y., Yano T., Oya K., Fujisawa J.-i., Hanaya M. (2015). Highly-efficient dye-sensitized solar cells with collaborative sensitization by silyl-anchor and carboxy-anchor dyes. Chem. Commun..

[32] Kumavat P.P., Sonar P., Dalal D.S. (2017). An overview on basics of organic and dye sensitized solar cells, their mechanism and recent improvements. Renew. Sustain. Energy Rev..

[33] Mishra A., Fischer M.K.R., Bäuerle P. (2009). Metal-free organic dyes for dye-sensitized solar cells: From structure: Property relationships to design rules. Angew. Chem. Int. Ed..

[34] Nalzala Thomas M.R., Kanniyambatti Lourdusamy V.J., Dhandayuthapani A.A., Jayakumar V. (2021). Non-metallic organic dyes as photosensitizers for dye-sensitized solar cells: A review. Environ. Sci. Pollut. Res..

[35] Ooyama Y., Harima Y. (2009). Molecular designs and syntheses of organic dyes for dye-sensitized solar cells. Eur. J. Org. Chem..

[36] Naik P., Su R., Elmorsy M.R., El-Shafei A., Adhikari A.V. (2018). New carbazole based dyes as effective co-sensitizers for DSSCs sensitized with ruthenium (II) complex (NCSU-10). J. Energy Chem..

[37] Bouchard J., Belletête M., Durocher G., Leclerc M. (2003). Solvatochromic properties of 2,7-carbazole-based conjugated polymers. Macromolecules.

[38] Damit E.F., Nordin N., Ariffin A., Sulaiman K. (2016). Synthesis of novel derivatives of carbazole-thiophene, their electronic properties, and computational studies. J. Chem..

[39] Elmorsy M.R., Badawy S.A., Salem K.E., Fadda A.A., Abdel-Latif E. (2023). New photosensitizers that are based on carbazoles and have thiophene bridges with a low bandgap do 32% better than N719 metal complex dye. J. Photochem. Photobiol. A.

[40] Venkateswararao A., Thomas K.R.J., Lee C.-P., Li C.-T., Ho K.-C. (2014). Organic dyes containing carbazole as donor and π-linker: Optical, electrochemical, and photovoltaic properties. ACS Appl. Mater. Interfaces.

[41] Soni S.S., Fadadu K.B., Vaghasiya J.V., Solanki B.G., Sonigara K.K., Singh A., Das D., Iyer P.K. (2015). Improved molecular architecture of D–π–A carbazole dyes: 9% PCE with a cobalt redox shuttle in dye sensitized solar cells. J. Mater. Chem. A.

[42] Zhang H., Iqbal Z., Chen Z.-E., Hong Y.-P. (2017). Effect of structural optimization on the photovoltaic performance of dithieno[3,2-b:2′,3′-d]pyrrole-based dye-sensitized solar cells. RSC Adv..

[43] Jiao Y., Mao L., Liu S., Tan T., Wang D., Cao D., Mi B., Gao Z., Huang W. (2018). Effects of meta or para connected organic dyes for dye-sensitized solar cell. Dye. Pigment..

[44] Zhang H., Iqbal Z., Chen Z.-E., Hong Y. (2018). Effects of various heteroatom donor species on the photophysical, electrochemical and photovoltaic performance of dye-sensitized solar cells. Electrochim. Acta.

[45] Duvva N., Giribabu L. (2020). Hexyl dithiafulvalene (HDT)-substituted carbazole (CBZ) D–π–A based sensitizers for dye-sensitized solar cells. New J. Chem..

[46] Tian L., Zhang X., Xu X., Pang Z., Li X., Wu W., Liu B. (2020). The planarization of side chain in carbazole sensitizer and its effect on optical, electrochemical, and interfacial charge transfer properties. Dye. Pigment..

[47] Qian X., Zhu Y.-Z., Chang W.-Y., Song J., Pan B., Lu L., Gao H.-H., Zheng J.-Y. (2015). Benzo[a]carbazole-Based Donor−π–Acceptor Type Organic Dyes for Highly Efficient Dye-Sensitized Solar Cells. ACS Appl. Mater. Interfaces.

[48] Su J., Chen Y., Wu Y., Ghimire R.P., Xu Y., Liu X., Wang Z., Liang M. (2017). New triarylamine organic dyes containing the 9-hexyl-2-(hexyloxy)-9H-carbazole for dye-sensitized solar cells. Electrochim. Acta.

[49] Wu Z.-S., Song X.-C., Liu Y.-D., Zhang J., Wang H.-S., Chen Z.-J., Liu S., Weng Q., An Z.-W., Guo W.-J. (2020). New organic dyes with varied arylamine donors as effective co-sensitizers for ruthenium complex N719 in dye sensitized solar cells. J. Power Sources.

[50] Thongkasee P., Thangthong A., Janthasing N., Sudyoadsuk T., Namuangruk S., Keawin T., Jungsuttiwong S., Promarak V. (2014). Carbazole-dendrimer-based donor−π–acceptor type organic dyes for dye-sensitized solar cells: Effect of the size of the carbazole dendritic donor. ACS Appl. Mater. Interfaces.

[51] Xiao Z., Chen B., Cheng X. (2021). Novel red light-absorbing organic dyes based on indolo[3,2-b]carbazole as the donor applied in co-sensitizer-free dye-sensitized solar cells. Materials.

[52] Periyasamy K., Sakthivel P., Ragavan I., Anbarasan P.M., Arunkumar A., Shkir M., Vidya C., Balasubramani V., Reddy V.R.M., Kim W.K. (2023). Design, synthesis, and optical and electrochemical properties of D–π–A type organic dyes with carbazole-based donor units for efficient dye-sensitized solar cells: Experimental and theoretical studies. J. Electron. Mater..

[53] Wagner K., Wagner P., Hasani F., Barnsley J.E., Gordon K.C., Lennert A., Guldi D.M., Officer D.L. (2021). Carbazole-substituted dialkoxybenzodithiophene dyes for efficient light harvesting and the effect of alkoxy tail length. Dye. Pigment..

[54] An J., Tian Z., Zhang L., Yang X., Cai B., Yu Z., Zhang L., Hagfeldt A., Sun L. (2021). Supramolecular Co-adsorption on TiO_2_ to enhance the efficiency of dye-sensitized solar cells. J. Mater. Chem. A.

[55] Saravana Kumaran T., Prakasam A., Vennila P., Parveen Banu S., Venkatesh G. (2021). New Carbazole-based organic dyes with various acceptors for dye-sensitized solar cells: Synthesis, characterization, dsscs fabrications and DFT study. Asian J. Chem..

[56] He J., Hua J., Hu G., Yin X.J., Gong H., Li C. (2014). Organic dyes incorporating a thiophene or furan moiety for efficient dye-sensitized solar cells. Dye. Pigment..

[57] Su J.-Y., Lo C.-Y., Tsai C.-H., Chen C.-H., Chou S.-H., Liu S.-H., Chou P.-T., Wong K.-T. (2014). Indolo[2,3-b]carbazole synthesized from a double-intramolecular Buchwald–Hartwig reaction: Its application for a dianchor dssc organic dye. Org. Lett..

[58] Han L., Zu X., Cui Y., Wu H., Ye Q., Gao J. (2014). Novel D–A–π–A carbazole dyes containing benzothiadiazole chromophores for dye-sensitized solar cells. Org. Electron..

[59] Ghasempour Nesheli F., Tajbakhsh M., Hosseinzadeh B., Hosseinzadeh R. (2020). Design, Synthesis and Photophysical Analysis of New Unsymmetrical Carbazole-Based Dyes for Dye-Sensitized Solar Cells. J. Photochem. Photobiol. A.

[60] El Fakir Z., Idrissi A., Habsaoui A., Bouzakraoui S. (2023). Small carbazole-based molecules as hole transporting materials for perovskite solar cells. J. Mol. Graph. Model..

[61] Liu Y., Zhang X., Li C., Tian Y., Zhang F., Wang Y., Wu W., Liu B. (2019). Energy-level control via molecular planarization and its effect on interfacial charge-transfer processes in dye-sensitized solar cells. J. Phys. Chem. C.

[62] Wang Y., Zhang G. (2022). Effect of molecular shape on the properties of indolo[3,2,1-jk]carbazole-based compounds. Eur. J. Org. Chem..

[63] Zhu Q., Zhang X., Pang Z., Wu W., Liu B. (2020). Molecular engineering of the alkyl chain in planar carbazole dyes toward efficient interfacial charge transfer processes. New J. Chem..

[64] Jang Y., Thogiti S., Lee K.-y., Kim J. (2019). Long-term stable solid-state dye-sensitized solar cells assembled with solid-state polymerized hole-transporting material. Crystals.

[65] Liu J., Li Y., Yong S., Arumugam S., Beeby S. (2019). Flexible printed monolithic-structured solid-state dye sensitized solar cells on woven glass fibre textile for wearable energy harvesting applications. Sci. Rep..

[66] Li D., Qin D., Deng M., Luo Y., Meng Q. (2009). Optimization the solid-state electrolytes for dye-sensitized solar cells. Energy Environ. Sci..

[67] Schmidt-Mende L., Grätzel M. (2006). TiO_2_ pore-filling and its effect on the efficiency of solid-state dye-sensitized solar cells. Thin Solid Films.

[68] Snaith H.J., Schmidt-Mende L. (2007). Advances in liquid-electrolyte and solid-state dye-sensitized solar cells. Adv. Mater..

[69] Ding I.K., Tétreault N., Brillet J., Hardin B.E., Smith E.H., Rosenthal S.J., Sauvage F., Grätzel M., McGehee M.D. (2009). Pore-filling of spiro-OMeTAD in solid-state dye sensitized solar cells: Quantification, mechanism, and consequences for device performance. Adv. Funct. Mater..

[70] Wu J., Lan Z., Lin J., Huang M., Huang Y., Fan L., Luo G. (2015). Electrolytes in dye-sensitized solar cells. Chem. Rev..

[71] Krüger J., Plass R., Cevey L., Piccirelli M., Grätzel M., Bach U. (2001). High efficiency solid-state photovoltaic device due to inhibition of interface charge recombination. Appl. Phys. Lett..

[72] Kim J.-Y., Kim J.Y., Lee D.-K., Kim B., Kim H., Ko M.J. (2012). Importance of 4-tert-butylpyridine in electrolyte for dye-sensitized solar cells employing SnO_2_ electrode. J. Phys. Chem. C.

[73] Yang L., Lindblad R., Gabrielsson E., Boschloo G., Rensmo H., Sun L., Hagfeldt A., Edvinsson T., Johansson E.M.J. (2018). Experimental and theoretical investigation of the function of 4-tert-butyl pyridine for interface energy level adjustment in efficient solid-state dye-sensitized solar cells. ACS Appl. Mater. Interfaces.

[74] Nazeeruddin M.K., Kay A., Rodicio I., Humphry-Baker R., Mueller E., Liska P., Vlachopoulos N., Graetzel M. (1993). Conversion of light to electricity by cis-X2bis(2,2'-bipyridyl-4,4'-dicarboxylate)ruthenium(II) charge-transfer sensitizers (X = Cl-, Br-, I-, CN-, and SCN-) on nanocrystalline titanium dioxide electrodes. J. Am. Chem. Soc..

[75] Haque S.A., Tachibana Y., Willis R.L., Moser J.E., Grätzel M., Klug D.R., Durrant J.R. (2000). Parameters influencing charge recombination kinetics in dye-sensitized nanocrystalline titanium dioxide films. J. Phys. Chem. B.

[76] Krüger J., Plass R., Grätzel M., Cameron P.J., Peter L.M. (2003). Charge transport and back reaction in solid-state dye-sensitized solar cells:  A study using intensity-modulated photovoltage and photocurrent spectroscopy. J. Phys. Chem. B.

[77] Yu Q., Wang Y., Yi Z., Zu N., Zhang J., Zhang M., Wang P. (2010). High-efficiency dye-sensitized solar cells: The influence of lithium ions on exciton dissociation, charge recombination, and surface states. ACS Nano.

[78] Juarez-Perez E.J., Leyden M.R., Wang S., Ono L.K., Hawash Z., Qi Y. (2016). Role of the dopants on the morphological and transport properties of spiro-MeOTAD hole transport layer. Chem. Mater..

[79] Salzmann I., Heimel G., Oehzelt M., Winkler S., Koch N. (2016). Molecular electrical doping of organic semiconductors: Fundamental mechanisms and emerging dopant design rules. Acc. Chem. Res..

[80] Xu B., Bi D., Hua Y., Liu P., Cheng M., Grätzel M., Kloo L., Hagfeldt A., Sun L. (2016). A low-cost spiro[fluorene-9,9′-xanthene]-based hole transport material for highly efficient solid-state dye-sensitized solar cells and perovskite solar cells. Energy Environ. Sci..

[81] Luo J., Xia J., Yang H., Chen L., Wan Z., Han F., Malik H.A., Zhu X., Jia C. (2018). Toward high-efficiency, hysteresis-less, stable perovskite solar cells: Unusual doping of a hole-transporting material using a fluorine-containing hydrophobic Lewis acid. Energy Environ. Sci..

[82] Hou Y., Du X., Scheiner S., McMeekin D.P., Wang Z., Li N., Killian M.S., Chen H., Richter M., Levchuk I. (2017). A generic interface to reduce the efficiency-stability-cost gap of perovskite solar cells. Science.

[83] Salbeck J., Yu N., Bauer J., Weissörtel F., Bestgen H. (1997). Low molecular organic glasses for blue electroluminescence. Synth. Met..

[84] Pudzich R., Fuhrmann-Lieker T., Salbeck J. (2006). Spiro compounds for organic electroluminescence and related applications. Emissive Materials Nanomaterials.

[85] Bailie C.D., Unger E.L., Zakeeruddin S.M., Grätzel M., McGehee M.D. (2014). Melt-infiltration of spiro-OMeTAD and thermal instability of solid-state dye-sensitized solar cells. Phys. Chem. Chem. Phys..

[86] Bach U., Lupo D., Comte P., Moser J.E., Weissörtel F., Salbeck J., Spreitzer H., Grätzel M. (1998). Solid-state dye-sensitized mesoporous TiO_2_ solar cells with high photon-to-electron conversion efficiencies. Nature.

[87] Puckyte G., Schmaltz B., Tomkeviciene A., Degbia M., Grazulevicius J.V., Melhem H., Bouclé J., Tran-Van F. (2013). Carbazole-based molecular glasses for efficient solid-state dye-sensitized solar cells. J. Power Sources.

[88] Degbia M., Schmaltz B., Bouclé J., Grazulevicius J.V., Tran-Van F. (2014). Carbazole based hole transporting materials for solid state dye sensitizer solar cells: Role of the methoxy groups. Polym. Int..

[89] Michaleviciute A., Degbia M., Tomkeviciene A., Schmaltz B., Gurskyte E., Grazulevicius J.V., Bouclé J., Tran-Van F. (2014). Star-shaped carbazole derivative based efficient solid-state dye sensitized solar cell. J. Power Sources.

[90] Xu B., Sheibani E., Liu P., Zhang J., Tian H., Vlachopoulos N., Boschloo G., Kloo L., Hagfeldt A., Sun L. (2014). Carbazole-based hole-transport materials for efficient solid-state dye-sensitized solar cells and perovskite solar cells. Adv. Mater..

[91] Bui T.-T., Shah S.K., Abbas M., Sallenave X., Sini G., Hirsch L., Goubard F. (2015). Carbazole-based molecular glasses as hole-transporting materials in solid state dye-sensitized solar cells. ChemNanoMat.

[92] Degbia M., Ben Manaa M., Schmaltz B., Berton N., Bouclé J., Antony R., Tran Van F. (2016). Carbazole-based hole transporting material for solid state dye-sensitized solar cells: Influence of the purification methods. Mater. Sci. Semicond. Process..

[93] Jung S., Kwon Y. (2022). Synthesis and characterization of hole transporting materials containing bis(carbazole) groups for solid-state dye-sensitized solar cells. Mol. Cryst. Liq. Cryst..

[94] Benhattab S., Nakar R., Rodriguez Acosta J.W., Berton N., Faure-Vincent J., Bouclé J., Tran Van F., Schmaltz B. (2018). Carbazole-based twin molecules as hole-transporting materials in dye-sensitized solar cells. Dye. Pigment..

[95] Degbia M., Schmaltz B., Van F.T., Melhem H., Bouclé J., Tomkeviciene A., Grazulevicius J.V. Comparative study of 2,7 versus 3,6 disubstituted carbazole as hole transporting materials in solid state DSSC. Proceedings of the 2012 International Semiconductor Conference Dresden-Grenoble (ISCDG).

[96] Wang L., Sheibani E., Guo Y., Zhang W., Li Y., Liu P., Xu B., Kloo L., Sun L. (2019). Impact of linking topology on the properties of carbazole-based hole-transport materials and their application in solid-state mesoscopic solar cells. Sol. RRL.

[97] Kong M., Kim K.S., Nga N.V., Lee Y., Jeon Y.S., Cho Y., Kwon Y., Han Y.S. (2020). Molecular weight effects of biscarbazole-based hole transport polymers on the performance of solid-state dye-sensitized solar cells. Nanomaterials.

[98] Nguyen V.N., Kwon Y. (2019). Synthesis of poly[n-(2-ethylhexyl)-3,6-carbazole-alt-aniline] copolymer and its potential as hole-transporting material to solid-state dye-sensitized solar cells. Mol. Cryst. Liq. Cryst..

[99] Ryu K.H., Oh D.H., Shin S., Kim M., Han Y.S. (2023). Effects of metal bis(trifluoromethanesulfonyl)imides as dopants of a hole-transporting tercarbazole compound on the photovoltaic performance of solid-state dye-sensitized solar cells. Mol. Cryst. Liq. Cryst..

[100] Leijtens T., Ding I.K., Giovenzana T., Bloking J.T., McGehee M.D., Sellinger A. (2012). Hole transport materials with low glass transition temperatures and high solubility for application in solid-state dye-sensitized solar cells. ACS Nano.

[101] Tomkeviciene A., Puckyte G., Grazulevicius J.V., Degbia M., Tran-Van F., Schmaltz B., Jankauskas V., Bouclé J. (2012). Diphenylamino-substituted derivatives of 9-phenylcarbazole as glass-forming hole-transporting materials for solid state dye sensitized solar cells. Synth. Met..

