# Supplementary material for: Advancements in Carbazole-Based Sensitizers and Hole-Transport Materials for Enhanced Photovoltaic Performance
Source: Molecules. 2024 Oct 25;29(21):5035. doi: 10.3390/molecules29215035 (PMC11547213; doi:10.3390/molecules29215035)
Supplement: Supplementary file 1 [file molecules-29-05035-s001.zip › molecules-3224295-supplementary.pdf]

Advancements in Carbazole-Based Sensitizers and Hole-Transport Materials for Enhanced Photovoltaic Performance

Ayagoz Ibrayeva, Urker Abibulla, Zulfiya Imanbekova, Bakhytzhan Baptayev, Robert J. O'Reilly and Mannix P. Balanay

Supporting Information

Table S1. A comprehensive characterization of the optical, thermal, electrochromic, and photovoltaic parameters of the carbazole-based hole-transport materials

HTM	HOMO (eV)	LUMO (eV)	Band gap (eV)	λ_{max} (soln) (nm)	T_g (°C)	T_m (°C)	T_{id} (°C)	Hole mobility ($cm^2 V^{-1} s^{-1}$)	TiO_2 thickness (µm)	Dye	CE	dopants	Area (cm^2)	J_{SC} (mA/cm^2)	V_{OC} (mV)	FF (%)	PCE (%)	Ref
H1	–4.95	–2.10	2.85	420[a]	65	97	388	4.40 x 10^{-4}	2 – 2.5	D102	Au	LiTFSI, tBP	0.18	2.63	630	32	0.54	[80]
H2	–5.12	–2.17	2.95	435[a]	145	–	441	3.30 x 10^{-3}	2 – 2.5	D102	Au	LiTFSI, tBP	0.18	9.47	690	53	3.44	[80]
H3	–	–	2.92	355[a]	98	–	378	–	2 – 2.5	D102	Au	LiTFSI, tBP	0.18	0.32	860	44	0.12	[81]
H4	–	–	2.95	375[a]	85	–	392	–	2 – 2.5	D102	Au	LiTFSI, tBP	0.18	6.32	680	41	1.75	[81]
H4	–5.43	–0.50	5.93	310[b]	–	–	–	1.19 x 10^{-4}	2	LEG4	Ag	LiTFSI, tBP	–	9.62	750	62	4.50	[83]
H5	–	–	–	420[a]	164	262	490	–	0.5 – 1	D102	Au	LiTFSI, tBP	0.18	8.85	740	34	2.23	[82]
H6	–	–	–	420[a]	175	308	480	–	0.5 – 1	D102	Au	LiTFSI, tBP	0.18	1.57	730	30	0.30	[82]
H7	–5.43	–0.50	5.93	310[b]	–	–	–	–	2	LEG4	Ag	LiTFSI, tBP	–	9.27	920	70	6.00	[83]
H8	--	--	--	372[a]	80	--	375	1.77 x 10^{-7}	–	D102	Ag	LiTFSI, tBP		4.41	760	48	1.60	[84]
H8	–	–	–	372[a]	68	–	370	–	2 – 2.5	D102	Au	LiTFSI, tBP	0.18	2.68	650	47	0.82	[85]
H8									2 – 2.5	D102	Au	LiTFSI, tBP		3.34	800	60	1.62[x]	[85]
H9	--	--	--	372[a]	55	--	398	2.87 x10^{-7}	–	D102	Ag	LiTFSI, tBP	0.09	5.25	750	46	1.81	[84]
H10	--	--	--	371[a]	66	--	336	1.18 x 10^{-7}	–	D102	Ag	LiTFSI, tBP	0.09	–	–	–	–	[84]
H11	–5.52	–2.26	3.26	301	–	–	–	–	–	N719	Pt	LiTFSI, tBP, MPII	0.25	4.9	590	80	2.30	[86]
H12	–5.61	–2.24	3.37	305	–	–	–	–	–	N719	Pt	LiTFSI, tBP, MPII	0.25	6.55	550	70	2.54	[86]
H13	–4.82	–	–	303, 372[a]	107	–	–	–	–	D102	Ag	LiTFSI, tBP	0.18	6.27	690	51	2.21	[87]

continuation of **Table S1**

HTM	HOMO (eV)	LUMO (eV)	Band gap (eV)	λ_{max} (soln) (nm)	T_g (°C)	T_m (°C)	T_{id} (°C)	Hole mobility ($cm^2 V^{-1} s^{-1}$)	TiO_2 thickness (μm)	Dye	CE	dopants	Area (cm^2)	J_{SC} (mA/cm^2)	V_{OC} (mV)	FF (%)	PCE (%)	Ref
H14	–4.81	–	–	302, 372[a]	66	–	–	–	–	D102	Ag	LiTFSI, tBP	0.18	0.86	590	38	0.20	[87]
H15	–4.80	–	–	300, 372[a]	47	–	–	–	–	D102	Ag	LiTFSI, tBP	0.18	0.21	660	34	0.05	[87]
H16	–4.7	–	–	–	100	258	–	–	2	D102	Au	–	0.18	0.95	590	32	0.18	[88]
H17	–5.1	–	–	–	99	223	–	–	2	D102	Au	–	0.18	1.57	760	29	0.34	[88]
H18	–	–	–	391[c]	95	–		2.23 x 10^{-4}	2	LEG4	Ag	LiTFSI, tBP	–	10.3	810	58	5.80	[89]
H19	–	–	–	416[c]	100	–		2.88 x 10^{-4}	2	LEG4	Ag	LiTFSI, tBP	–	10.3	880	75	6.80	[89]
H20	–5.11	–2.27	2.84	302[d]	–	–	–	4.33	–	N719	Pt	LiTFSI, tBP, MPII	0.25	9.54	590	67	3.79[y]	[90]
H20	–5.11	–2.27	2.84	302[d]	–	–	–	1.50	–	N719	Pt	LiTFSI, tBP, MPII	0.25	7.53	566	64	2.73[z]	[90]
H21	–5.19	–2.28	2.91	309	–	–	442	0.7 x 10^{-5}	–	N719	Pt	LiTFSI, tBP, MPII	0.25	3.76	538	74	1.5	[91]
H22	–	–	–	–	–	–	–	–	–	N719	Pt	LiTFSI, MPII	0.25	2.48	627	55	0.85	[92]
H22	–	–	–	–	–	–	–	–	–	N719	Pt	AgTFSI, MPII	0.25	1.30	661	58	0.50	[92]
H22	–	–	–	–	–	–	–	–	–	N719	Pt	$Zn(TFSI)_2$, MPII	0.25	1.71	655	56	0.63	[92]
H22	–	–	–	–	–	–	–	–	–	N719	Pt	$Mg(TFSI)_2$, MPII	0.25	4.02	611	61	1.49	[92]
H23	–4.98	–1.93	2.96	393[d]	59	132	–	1 x 10^{-5}	2	Z907	Ag	LiTFSI, tBP	3	–	–	–	0.50	[93]
H23	–	–	–	–	–	–	–	1 x 10^{-5}	2	Z907	Ag	TBPA	3	–	–	–	2.30	[93]
H23	–	–	–	–	–	–	–	–	6	Z907	Ag	TBPA	3	5.38	720	58	2.26	[93]
H24	–4.82	–1.88	2.94	420[a]	65	201	362	–	2	D102	Au	LiTFSI, tBP	0.18	2.63	630	32	0.54	
H25	–4.92	–1.85	3.07	403[a]	69	145	388	–	2	D102	Au	LiTFSI, tBP	0.18	0.75	573	28	0.12	
H26	–4.86	–1.92	2.94	415[a]	89	174	400	–	2	D102	Au	LiTFSI, tBP	0.18	1.72	531	35	0.32	

The solvent used for the determination of λ_{max} is [a] tetrahydrofuran, [b] dichloromethane, [c] toluene, and [d] dichlorobenzene.

[x] HTM was purified using a sublimation technique. Data from [y] high and [z] low molecular weight polymer.
